# Transcriptional profiling identifies differentially expressed genes in developing turkey skeletal muscle

**DOI:** 10.1186/1471-2164-12-143

**Published:** 2011-03-08

**Authors:** Kelly RB Sporer, Robert J Tempelman, Catherine W Ernst, Kent M Reed, Sandra G Velleman, Gale M Strasburg

**Affiliations:** 1 Department of Food Science and Human Nutrition, Michigan State University, East Lansing, Michigan, 48824, USA; 2 Department of Animal Science, Michigan State University, East Lansing, Michigan, 48824, USA; 3 Department of Veterinary and Biomedical Sciences, University of Minnesota, St. Paul, Minnesota, 55108, USA; 4 Department of Animal Sciences, The Ohio State University/Ohio Agricultural Research and Development Center, Wooster, Ohio, 44691, USA

## Abstract

**Background:**

Skeletal muscle growth and development from embryo to adult consists of a series of carefully regulated changes in gene expression. Understanding these developmental changes in agriculturally important species is essential to the production of high quality meat products. For example, consumer demand for lean, inexpensive meat products has driven the turkey industry to unprecedented production through intensive genetic selection. However, achievements of increased body weight and muscle mass have been countered by an increased incidence of myopathies and meat quality defects. In a previous study, we developed and validated a turkey skeletal muscle-specific microarray as a tool for functional genomics studies. The goals of the current study were to utilize this microarray to elucidate functional pathways of genes responsible for key events in turkey skeletal muscle development and to compare differences in gene expression between two genetic lines of turkeys. To achieve these goals, skeletal muscle samples were collected at three critical stages in muscle development: 18d embryo (hyperplasia), 1d post-hatch (shift from myoblast-mediated growth to satellite cell-modulated growth by hypertrophy), and 16wk (market age) from two genetic lines: a randombred control line (RBC2) maintained without selection pressure, and a line (F) selected from the RBC2 line for increased 16wk body weight. Array hybridizations were performed in two experiments: Experiment 1 directly compared the developmental stages within genetic line, while Experiment 2 directly compared the two lines within each developmental stage.

**Results:**

A total of 3474 genes were differentially expressed (false discovery rate; FDR < 0.001) by overall effect of development, while 16 genes were differentially expressed (FDR < 0.10) by overall effect of genetic line. Ingenuity Pathways Analysis was used to group annotated genes into networks, functions, and canonical pathways. The expression of 28 genes involved in extracellular matrix regulation, cell death/apoptosis, and calcium signaling/muscle function, as well as genes with miscellaneous function was confirmed by qPCR.

**Conclusions:**

The current study identified gene pathways and uncovered novel genes important in turkey muscle growth and development. Future experiments will focus further on several of these candidate genes and the expression and mechanism of action of their protein products.

## Background

Hyperplasia and hypertrophy are key processes in myogenesis in the domestic turkey. It is well established that the number of muscle fibers is determined embryonically [1], as myoblasts, originating as somites, migrate to the appropriate site of muscle formation, then proliferate during the process of hyperplasia. These myoblasts then withdraw from the cell cycle, fuse to form multi-nucleated myotubes, and differentiate with commencement of muscle-specific protein expression. After the actual number of muscle fibers is determined during hyperplasia, skeletal muscle stem cells, satellite cells located beneath the basal lamina of the muscle fibers, are activated and fuse with muscle fibers to contribute their nuclei, resulting in a further increase in DNA that directly leads to an increase in protein synthesis. This post-hatch increase in muscle fiber size (hypertrophy) is responsible for the majority of overall muscle mass accretion [2,3].

All of these events are precisely temporally and spatially regulated by transcription factors, growth factors, and interactions with the extracellular matrix (ECM). The transcription factors Pax3 and Pax7 are responsible early on for determination of cells entering the myogenic program as well as the activation and survival of satellite cells [4,5]. Myogenic regulatory factors (MRFs), including the four basic helix-loop-helix transcription factors Myf5, myogenin, MRF4, and MyoD, are pivotal in regulating genes involved with commitment of proliferating somatic cells to the myogenic lineage and subsequent differentiation [6]. Myocyte enhancer factors (MEFs) comprise another group of transcription factors that work in conjunction with the MRFs to ensure appropriate expression of muscle-specific proteins during differentiation. Binding sites for MEF2s have been discovered in the promoters of many crucial skeletal muscle structural genes [7]. These groups of transcription factors also regulate each other [6,7]. Furthermore, the activation, proliferation, and differentiation of satellite cells are regulated by various growth factors, including insulin-like growth factors (IGF), fibroblast growth factor 2 (FGF2), platelet-derived growth factors (PDGF), hepatocyte growth factors, (HGF), epidermal growth factors (EGF), and transforming growth factor-β (TGF-β) [8]. Satellite cell response to the growth factors IGF and FGF2 is different between fast-growing and slow-growing breeds of domestic turkeys [9].

A genetic model has been developed and maintained to study the effects of growth selection in turkeys. This model consists of two lines: a randomly bred control line of turkeys (RBC2), maintained without selection pressure and representing a commercial turkey from 1967 [10], and a unique line genetically derived from the RBC2 birds and selected for over 40 generations solely for 16-wk body weight (F) [11]. The F line turkeys gain body weight faster and yield significantly heavier breast muscles than the RBC2 birds [12]. In addition, satellite cells from F turkeys have higher proliferation and differentiation rates than those from RBC2 turkeys [13]. Growth comparisons between these two lines are not confounded by selection for other characteristics such as disease resistance, reproductive efficiency, or behavioral traits, as are commercial turkeys.

Many gene expression differences between these two lines have been identified *in vitro *using isolated satellite cells. The MRFs *MyoD *and *myogenin *are differentially expressed between F and RBC2 lines in embryonic pectoralis major muscle and cultured satellite cells [14]. In addition, proteoglycans in the extracellular matrix that interact with growth factors important for proliferation and differentiation of satellite cells exhibit differential expression between the two lines [15,16]. These cell culture studies have been pivotal for understanding how satellite cells contribute to muscle growth.

To build on this work, we developed a microarray approach that enabled the simultaneous analysis of skeletal muscle gene transcripts at specific time points *in vivo*. Previously, our group created and characterized a skeletal muscle-specific microarray, the Turkey Skeletal Muscle Long Oligonucleotide (TSKMLO) array, with the purpose of screening the skeletal muscle transcriptome for candidate genes critical in growth, proliferation, differentiation, and overall development [17,18]. Briefly, cDNA libraries were constructed and sequenced using turkey Pectoralis major muscle collected at three stages crucial to development. From these libraries, oligonucleotides representing 5257 putative transcripts as well as oligonucleotides representing unique sequences to control for nonspecific hybridization and template quality were designed and spotted, resulting in the 6K skeletal muscle-specific oligonucleotide microarray. Assessment of this platform using dye-swap experiments, evaluation of control probe signals, and qPCR validation of results established this array as a valid and valuable tool for functional genomics studies. The aims of the current study were to utilize this microarray to elucidate functional pathways of genes responsible for key events in turkey skeletal muscle development and to identify differences in temporal gene expression between two genetic lines.

## Methods

### Animals and Tissue Collection

Animals used in this study were domestic turkeys maintained at the Poultry Research Center at The Ohio Agricultural Research and Development Center located in Wooster, OH under AGACUC protocol #04-AG007. These turkeys were from two genetic lines: RBC2, a randomly bred control line representative of a 1967 commercial turkey [10,19], and F, a subline genetically selected from the RBC2 line for increased 16wk body weight [11]. The birds were euthanized immediately prior to tissue harvest at one of three developmental stages: 18 d embryo (18de; hyperplasia), 1 d post hatch (1d; shift from myoblast-mediated growth to satellite cell modulated growth by hypertrophy), and 16 wk old birds (16wk; approximate age of commercial slaughter). Pectoralis major muscle was excised [17], snap frozen in liquid nitrogen, and stored at -80°C until shipment on dry ice to Michigan State University (MSU), where samples were stored at -80°C until RNA extraction.

### RNA Extraction

Total RNA was isolated from skeletal muscle tissue using TRIReagent (Molecular Research Center, Inc., Cincinnati, OH) as per manufacturer instructions. All samples were treated with DNA-free™ DNase (Ambion, Inc., Austin, TX) to remove any contaminating genomic DNA. Sample integrity was confirmed using an Agilent 2100 Bioanalyzer (Santa Clara, CA) that determined RNA Integrity Number (RIN). All samples used for microarray analysis had an RIN > 8.0.

### Microarray Experimental Design

The TSKLMO array was designed and created based on sequences from cDNA libraries constructed at crucial stages in muscle development [18]. Description of this platform can be found on the National Center for Biotechnology Information (NCBI) Gene Expression Omnibus (GEO) database [GEO: GPL9788]. Experiment 1 (Additional File 1, Figure S1a) was designed to directly compare the three developmental stages, 18de vs. 1d or 1d vs. 16wk, for each of the genetic lines. Experiment 2 (Additional File 1, Figure S1b) was designed to directly compare the two genetic lines, F vs. RBC2, at each of the developmental stages. Each Experiment contained 10 arrays per comparison; therefore, Experiment 1 contained 40 arrays, and Experiment 2 contained 30 arrays. Birds were randomly assigned to array, and hybridizations were performed in random order.

### RNA Amplification and Microarray Hybridization

Total RNA for microarray analysis was amplified and dye-coupled in preparation for hybridization using the Amino Allyl MessageAmp™ II aRNA Amplification Kit (Ambion, Inc.) per manufacturer instructions. All microarray preparation and hybridization procedures were performed as previously described [17]. Briefly, all microarrays were UV-crosslinked and pre-hybridized prior to hybridization. Fragmented, Cy3-coupled aRNA was mixed with its Cy5-coupled partner, and the mixtures were hybridized to oligonucleotide microarrays for 18 h in a GeneTac Hybridization Station (Genomic Solutions, Ann Arbor, MI). Arrays were then rinsed, dried by centrifugation, and immediately scanned with a GenePix 4000B scanner (Molecular Devices, Sunnyvale, CA). Image analysis was performed using GenePix Pro 6.0 software, and spot intensities were exported as GPR files for statistical analysis. Fluorescence intensity data were not background-corrected in accordance with recent recommendations [20,21] but were normalized for dye intensity bias using the LOESS procedure of the normalizeWithinArray function of the Bioconductor R software LIMMA [22].

### Statistical Analysis

The two sets of experiments were analyzed separately. For the first experiment, in which 1d was directly compared against the other two stages within arrays and within lines, all microarray data were expressed as log_2 _ratios of each stage with 1d for each array. These log_2 _ratios were analyzed using a linear mixed model based on an overall intercept, the effect of dye assignment for samples deriving from stage 1d, effects of the other two stages specified as numerator terms in the log_2 _ratios, effect of line, and the random effects of the animal assigned to stage 1d and its interaction with the other two stages to serve as the experimental error term for stage as each oligonucleotide was spotted twice on an array. For this experiment, the comparisons of interest included all stages against each other within each of the two lines, as well as overall differences between the three stages.

For the second experiment, in which lines were compared directly against each other within arrays and within stages, all data were expressed as log_2 _ratios of F over RBC2 for each array. These log_2 _ratios were analyzed using a linear mixed model based on an overall intercept, the dye assignment for samples deriving from line RBC2, stage effects, the effect of line, and the random effect of the animal derived from the RBC2 line. The random effect could have been also arbitrarily chosen as the animal derived from the F line, but, in either case, this specification ensured that replication was based on the number of animals and not on the number of spots per gene per array. In this experiment, the comparisons of interest were overall line differences and line differences within each stage. SAS PROC MIXED was used for both analyses given the linear mixed model specifications for both experiments [23]. Statistical significance was based on the estimated false discovery rates (FDR) [24].

Raw Cy5 intensities, raw Cy3 intensities, LOESS-normalized log_2 _Cy5:Cy3 ratios, and LOESS-normalized log_2 _average intensities (A), as well as original GPR files for all arrays, were submitted to the National Center for Biotechnology Information (NCBI)'s Gene Expression Omnibus (GEO) [GSE19585]. Control probes were also evaluated for both Experiments 1 and 2. Results examining the fluorescence intensities of probes designed to contain an increasing number of mismatched bases as well as scrambled sequence oligos that served as negative controls indicated that hybridization was specific in these experiments. These data have been previously presented [17].

### Gene annotation and functional analysis

After statistical analysis, genes were sorted by FDR, and the top 200 genes differentially expressed by overall effect of development (Experiment 1) and all genes differentially expressed by overall effect of genetic line (Experiment 2) were investigated by subjecting oligonucleotide sequences to an NCBI BLAST search and choosing best matches for accession number and putative annotation. In addition, the top 30 up-regulated and down-regulated genes in Experiment 1 and all differentially expressed genes in Experiment 2 in each comparison: F 18de:1d, F 1d:16wk, RBC2 18de:1d, and RBC2 1d:16wk were annotated in the same manner. These lists were then probed for patterns in expression using the Ingenuity Pathways Analysis software (IPA; Redwood City, CA).

### Quantitative real-time PCR confirmation

Twenty-eight genes were chosen by function of interest and high differential expression by microarray analysis for confirmation of those results by quantitative real-time PCR (qPCR). Primers (Table 1) were designed using Primer Express 3.0 software (Applied Biosystems, Foster City, CA) and synthesized by Operon Inc. (Huntsville, AL). Sample RNAs (2 μg) were reverse transcribed to cDNA using Superscript III (Invitrogen, Carlsbad, CA) and quantified with a Nanodrop ND-1000 spectrophotometer (Thermo Fisher Scientific, Inc., Wilmington, DE). Reactions were run using 10 ng cDNA, 300 nM primer mix, and POWR SYBR Green Master Mix in a 7700 thermocycler system (Applied Biosystems). Data were analyzed by the 2^-ΔΔCt ^method [25] using *hypoxanthine phosphoribosyltransferase (HPRT) *as a reference gene and the RBC2, 18de stage as the calibrator; i.e., RBC2, 18de expression is set to "1.0," and all other results are shown as a fold change relative to this control. Statistical analysis was performed using a mixed model and Tukey-Kramer test (SAS, Cary, NC).

**Table 1:** Primer information for genes selected for confirmation by qPCR.

Gene	GenBank Accession #	Forward primer (5'→3')	Reverse primer (5'→3')	Amplicon length, bp	Tm,°C
*Activin IIB Receptor*	NM_204317	GAGGAGCGCATTGCTCAGAT	GACAAGGCAGTCCGAGGTAGTG	60	83
*Beta parvin*	XM_416459	CCAGTCCATACAACTCGCCTTT	CTGTGGTAATACAGCTCACTGGATCT	71	77
*BTB domain containing 11*	XM_001234770	TCTCAAGATGCTGATTTCTGTTTGT	GTATCGACACATCACTAGAGGAAAGATC	74	75
*Calmodulin 1*	NM_001110364	GGCTGGATACACTTGGTGCAT	GGATCTGATGACCCAGGAAGTT	68	80
*Calreticulin*	AY393845	CGCCCTGACAACACCTATGA	CTCCAGGCTCCCCGATTC	63	82
*Caspase 3*	NM_204725	GGAACACGCCAGGAAACTTG	TCTGCCACTCTGCGATTTACA	63	78
*Collagen, type VI, alpha 1 (COL6A1)*	NM_205107	TTCCATTGGTGCTCTTGCTATG	TTTGGGATGATGGCGATACC	79	78
*Death-associated Protein (DAP)*	NM_001031003	TGGGCAGCTCTACTCTATGTGTTC	CAAGAGCAGTTGTTTCTAACTCAAAAG	79	78
*Fibroblast growth factor 2 (FGF2)*	NM_205433	CAATCAAAGGCGTAAGTGCAAA	GCCAGCAATCTGCCATCCT	61	80
*Gelsolin*	NM_204934	GCTTACCCCCCTCGTCTTTT	TGAGTCAGATCTCCAGGGACTTC	80	80
*Glypican-1*	AY551002	CCAGAGGTGGAGGTGGACAT	TCATGATCTTCAGCTGCATGATT	73	80
*Growth Arrest-Specific 2 (GAS2)*	XM_420902	TGATCCTTTCTGTTCCTGACAAAG	GGGAGAGGAAAAGGCAACCT	75	80
*Hypoxanthine phosphoribosyltransferase 1 (HPRT)*	NM_204848	CACAAGAAGCAGCCAGTTACAGTATC	CCCAGATTCCTTTTTACAGCAATAA	92	80
*Matrix Gla protein (MGP)*	NM_205044	GCACAATGCGTGCTCTCATC	AGCGGCCATGACCAAGAC	56	84
*Met proto-oncogene*	NM_205212	CAACAATCTTCTCCACTTTCATTGG	ATAGGGAGCAACACACTTCACGTT	83	77
*Myostatin*	AF019625	CACCTGGTACACCAAGCAAATC	TTGGTGGGTGTGCAGCAA	59	83
*Nebulin-related anchoring protein (NRAP)*	XM_002194458	GGCGTACCTTCGGGAAATG	GCAGAATTTCCGAAGCATCAG	122	82
*Nestin*	NM_205033	GCCCCGCGACAATCG	TGTTACAAACCAAAGGGAAATGG	71	83
*5'-nucleotidase, cytosolic III (NT5C3)*	NM_204436	TGTGGAGTGGGAAAGTCATACTGTT	CCTGTTGGTAAGAGGTTGAGAAACT	77	77
*Plastin 3*	NM_001006431	TGCTGAGGGCACAGCTACAC	CAACTGAACCAAGATGCATACAAAA	67	78
*Platelet-derived growth factor A (PDGFA)*	AB031021	TGCAACACCAGCAGTGTGAA	TTTGCCACCTTGACACTTCTGT	65	81
*Q3_Reed_1DPH_cDNA_08_B09_130 (Unknown)*	EH286878	CCCTGTGCTCATCCTATAAAGCA	CCACGATGCAGCAGGTGAT	61	82
*Spondin 2*	XM_420847	CGTTTTGTAGTTCTTCACCCTGCTA	TCACATTCTGTTTCTTCTTCCAGACT	71	77
*Syndecan-4*	AY852251	GCCAACAGCAGCATCTTTGA	CAACTGCTCCTCCTGCAATG	64	79
*Troponin C type 1 (slow) (TNNC1)*	NM_205133	AGATGGACAGCCCGAATCTCT	TGGGACCAAGGAGCTGATG	60	82
*Troponin I type 1 (skeletal, slow) (TNNI1)*	XM_419242	TCTGCCCTCTTTGCACCATT	GTTGTCCACGTGGTTCATCTCA	58	82
*Troponin T type 2 (cardiac)(TNNT2)*	NM_205449	GGCTCAGCCATCAGATGCA	CAGCAGAGCCCTGGCATAG	54	85
*Troponin T type 3 (skeletal, fast) (TNNT3)*	NM_204922	CCCGTGCCTCAGTGATAACTAAA	AGAAGAAAAGCAGCAGCAATAGC	68	78
*Versican*	NM_204787	CAGGATCATTTGTTTGCGGTTA	TCCTGATTCTTCCGCAAGTCA	60	80

## Results

### Differential expression of skeletal muscle genes

In Experiment 1, a total of 3474 genes were differentially expressed between at least two developmental stages across both genetic lines (FDR < 0.001; Table 2). Of these, 2544 genes were significantly affected for the RBC2 birds, whereas 2248 were significantly affected for the F birds (Table 2). A greater number of genes were up-regulated in the earlier developmental stage of direct comparisons (i.e., 18de in the 18de:1d comparison or 1d in the 1d:16wk comparison) as compared to those that were down-regulated, and this observation was more pronounced in the RBC2 line than the F line. The top 30 down- and up-regulated genes in each comparison are listed in Additional File 2, Table S1. A much smaller number of genes were significantly affected by genetic line in Experiment 2 (Table 2). Only 16 genes were differentially expressed by overall effect of line at an FDR < 0.10. When considering the effect of line within each developmental stage rather than overall effect across stages, the highest number of genes (63) were affected at the 18de developmental stage (RBC2:F; 39 up-regulated, 24 down-regulated). At 1d post-hatch, 20 genes were differentially expressed (5 up, 10 down), and at 16wk, 8 genes were differentially expressed (4 up, 4 down). The annotations for these genes are detailed in Additional File 3, Table S2.

**Table 2:** Breakdown of differentially expressed genes as affected by skeletal muscle development (Experiment 1) and by turkey genetic line (Experiment 2).

Experiment 1	Overall effect		
FDR < 0.001	3474		
RBC2	18de:1d	1d:16wk	Total
**Up**	586	1066	1652
**Down**	463	429	892
**Total**	1049	1495	
**F**	**18de:1d**	**1d:16wk**	**Total**

**Up**	455	746	1201
**Down**	345	702	1047
**Total**	800	1448	

**Experiment 2**	**Overall effect**		
**FDR < 0.10**	16		
**18de**	**RBC2:F**		

**Up**	39		
**Down**	24		
**Total**	63		
**1d**	**RBC2:F**		

**Up**	5		
**Down**	15		
**Total**	20		
**16wk**	**RBC2:F**		

**Up**	4		
**Down**	4		
**Total**	8		

### Functional and pathway analysis

After differentially expressed oligos on the TSKMLO array were annotated, lists of accession numbers were subjected to IPA analysis, and Networks (which involve regulatory relationships), Functions (which involve biological and disease processes), and Canonical Pathways (which involve well-characterized cell signaling and metabolic pathways) were considered when investigating functional groups of genes differentially expressed in turkey skeletal muscle during development. These results for Experiment 1 are shown separately for the RBC2 line and for the F line (Table 3). Experiment 2 results are shown in Table 4; developmental stages were grouped together because of the small number of differentially expressed genes.

**Table 3:** Top five Networks, Functions, and Canonical Pathways from IPA analysis for Experiment 1 for RBC2 and F lines.

	RBC2	F
**Networks**	Tissue Development, Cellular Growth and Proliferation, Skeletal and Muscular System Development and Function	Tissue Development, Skeletal and Muscular System Development and Function, Tissue Morphology
	
	Genetic Disorder, Skeletal and Muscular Disorders, Gene Expression	Cellular Development, Cell Cycle, Gene Expression
	
	Cancer, Cell Death, Cellular Assembly and Organization	Cell Cycle, Infectious Disease, Inflammatory Disease
	
	Gene Expression, Cellular Growth and Proliferation, Endocrine System Development and Function	Cellular Development, Cellular Growth and Proliferation, Respiratory System Development and Function
	
	Cell-To-Cell Signaling and Interaction, Cellular Growth and Proliferation, Cancer	Connective Tissue Development and Function, Organismal Development, Skeletal and Muscular System Development and Function

**Functions**	Genetic Disorder	Cancer
	
	Skeletal and Muscular Disorders	Skeletal and Muscular System Development and Function
	
	Cardiovascular System Development and Function	Tissue Morphology
	
	Skeletal and Muscular System Development and Function	Tissue Development
	
	Tissue Development	Small Molecule Biochemistry

**Canonical Pathways**	Calcium Signaling	Calcium Signaling
	
	Hepatic Fibrosis/Hepatic Stellate Cell Activation	Glycolysis/Gluconeogenesis
	
	Methionine Metabolism	Urea Cycle and Metabolism of Amino Groups
	
	Pyruvate Metabolism	Tight Junction Signaling
	
	Tight Junction Signaling	Arginine and Proline Metabolism

**Table 4:** Top Networks, Functions, and Canonical Pathways from IPA analysis for Experiment 2.

Networks	Cancer, Cell Morphology, Dermatological Diseases and Conditions
	Cell Signaling, DNA Replication, Recombination, and Repair, Nucleic Acid Metabolism
	
	Cell Cycle, Embryonic Development, Connective Tissue Development and Function
	
	Cancer, Reproductive System Disease, Hematological Disease

**Functions**	Cellular Assembly and Organization
	
	Cancer
	
	Cellular Movement
	
	Cell Cycle
	
	Renal and Urological Disease

**Canonical Pathways**	PI3K/AKT Signaling
	
	One Carbon Pool by Folate
	
	N-Glycan Degradation
	
	Biosynthesis of Steroids
	
	Urea Cycle and Metabolism of Amino Groups

### qPCR confirmation of differentially expressed genes

Genes in the categories of ECM function, cell death, and skeletal muscle development and function, along with several genes with miscellaneous functions, were selected for qPCR confirmation of microarray results. In addition, genes common to both Experiments 1 and 2 were selected for confirmation.

**Figure 1:**
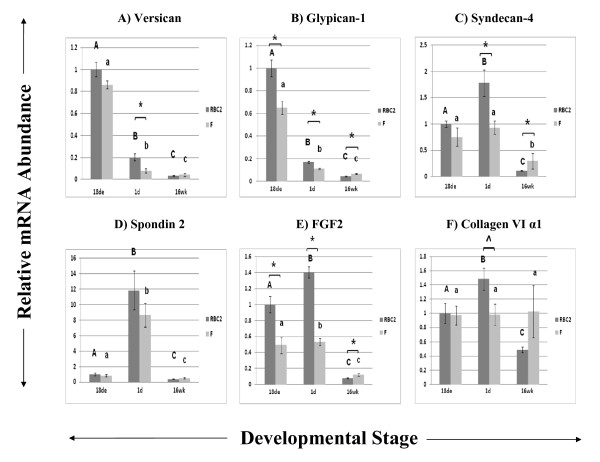
**Relative expression as determined by quantitative real-time RT-PCR of genes related to extracellular matrix (ECM) function**. Different letters (uppercase) represent differences between developmental stages for the RBC2 line; different letters (lowercase) represent differences between developmental stages for the F line (P ≤ 0.05). * represents differences between the two genetic lines, RBC2 and F (P ≤ 0.05); ^ represents differences between the two lines (0.05 < P < 0.10).

Figure 1 shows results for *versican *(1A), *glypican-1 *(1B), *syndecan-4 *(1C), *spondin 2 *(1D), *fibroblast growth factor 2 (FGF2*; 1E), and *collagen VI α1 *(*COL6A1*; 1F), which are all involved in ECM regulation. The *COL6A1 *gene was also common between Experiments 1 and 2. *Versican *and *glypican-1 *appeared to be expressed predominantly at the 18de stage when compared to later stages in development. Interestingly, *spondin 2 *was expressed almost exclusively at the 1d stage, during muscle hypertrophy. Several differences between the RBC2 and F lines were observed by qPCR analysis that were not detected with the significance threshold used for the microarray analysis. Most notably, *FGF2 *expression at the 18de stage was twice as high in the RBC2 line compared to that of the F line and nearly 3 times greater at the 1d stage in the RBC2 line compared to that of the F line.

**Figure 2:**
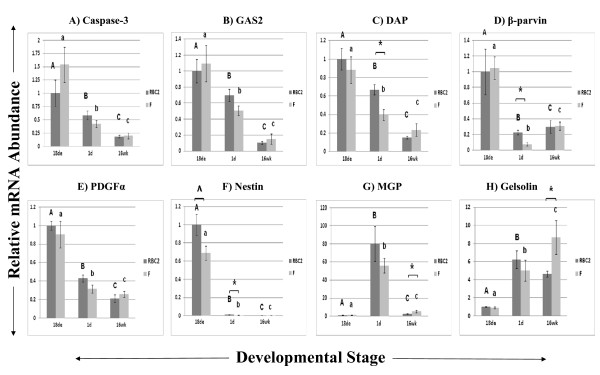
**Relative expression as determined by quantitative real-time RT-PCR of genes related to cell death/apoptosis**. Different letters (uppercase) represent differences between developmental stages for the RBC2 line; different letters (lowercase) represent differences between developmental stages for the F line (P ≤ 0.05). * represents differences between the two genetic lines, RBC2 and F (P ≤ 0.05); ^ represents differences between the two lines (0.05 < P < 0.10).

Figure 2 shows results for genes involved in cell death/apoptosis as determined by IPA: *caspase-3 *(2A), *growth arrest-specific 2 (GAS2*; 2B), *death-associated protein (DAP*; 2C), *β-parvin *(2D), *platelet-derived growth factor α (PDGFα*; 2E), *nestin *(2F), *matrix γ-carboxyglutamic acid (GLA) protein (MGP*; 2G), and *gelsolin *(2H). The gene *β-parvin *was also common to both Experiments 1 and 2. *Caspase-3, GAS2, DAP*, and *β-parvin *all appeared to be down-regulated as muscle develops. *Nestin *was highly expressed at the 18de stage with greater than 100 times the expression of subsequent developmental stages. The *MGP *gene, however, was expressed principally at the 1d stage at 80.28 and 55.38 times the 18de expression in RBC2 and F lines, respectively. *Gelsolin *expression appeared to increase with muscle maturation and peak at the 16wk time point.

**Figure 3:**
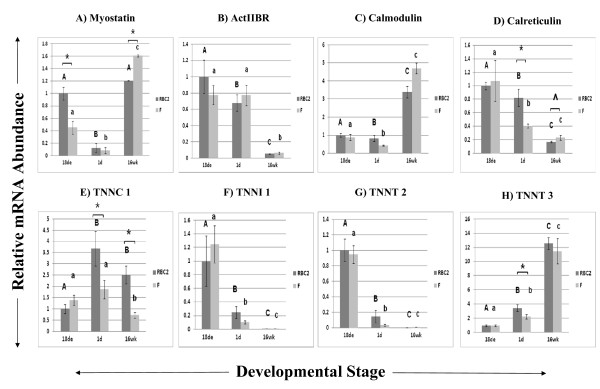
**Relative expression as determined by quantitative real-time RT-PCR of genes related to calcium regulation/muscle function**. Different letters (uppercase) represent differences between developmental stages for the RBC2 line; different letters (lowercase) represent differences between developmental stages for the F line (P ≤ 0.05). * represents differences between the two genetic lines, RBC2 and F (P ≤ 0.05); ^ represents differences between the two lines (0.05 < P < 0.10).

Genes important to skeletal muscle growth and development and calcium signaling are shown in Figure 3. These are *myostatin *(3A), *Activin IIB receptor (ActIIBR*; 3B), *calmodulin *(3C), *calreticulin *(3D), and isotypes of *troponin*: *TNNC1 *(3E), *TNNI1 *(3F), *TNNT2 *(3G), and *TNNT3 *(3H). *Myostatin *expression dipped at 1d posthatch, when hypertrophy occurs in muscle; interestingly, its expression was higher in the RBC2 line at 18de but was higher in the F line by 16wk of development. In conjunction, the myostatin receptor *ActIIBR *was highly down-regulated at 16wk (18de expression was 18.69 and 10.95 times greater in RBC2 and F lines, respectively), when *myostatin *expression is high. Relative *calmodulin *and *calreticulin *expression tended to respond in opposite directions with *calmodulin *expression highest at the 16wk stage and *calreticulin *highest at 18de and decreasing as muscle matures. Of the *troponins*, *TNNI1 *and *TNNT2 *exhibited similar profiles with predominant expression at 18de that decreased substantially at the other stages. Interestingly, *TNNT3 *expression appeared to be regulated in the opposite direction of *TNNT2*.

**Figure 4:**
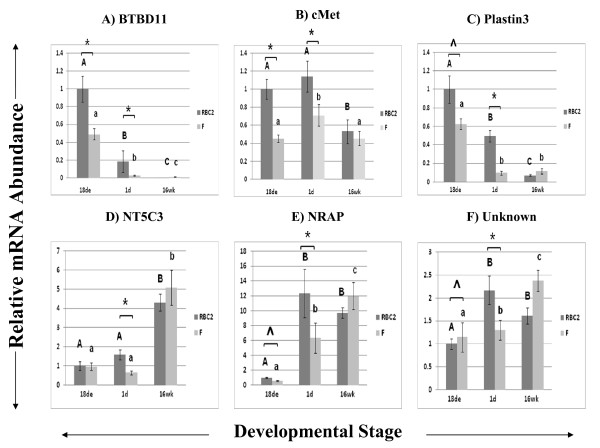
**Relative expression as determined by quantitative real-time RT-PCR of genes common to Experiments 1 and 2 and genes without known functions**. Unknown = Q3_Reed_1DPH_cDNA_08_B09_130. Different letters (uppercase) represent differences between developmental stages for the RBC2 line; different letters (lowercase) represent differences between developmental stages for the F line (P ≤ 0.05). * represents differences between the two genetic lines, RBC2 and F (P ≤ 0.05); ^ represents differences between the two lines (0.05 < P < 0.10).

The expression profiles of several other genes that were common to both Experiments 1 and 2 but did not easily fall into a functional category, along with one gene with unknown function, are shown in Figure 4. These genes included: *BTBD11 *(4A), *cMet *(4B), *plastin3 *(4C), *5'-nucleotidase, cytosolic III (NT5C3*; 4D), and *nebulin-related anchoring protein (NRAP*; 4E). In addition, a gene with no known annotation was chosen from the top 40 genes differentially expressed by overall effect of development (FDR < 1.0 × 10^-13^) and was subsequently coded Unknown (4F). Expression of these genes was variable although line differences were observed at 1d for all genes. Expression of *NRAP *was dramatically up-regulated at 1d and 16wk when compared to 18de. Expression profiles for these 28 genes confirmed that microarray results were repeatable.

## Discussion

Muscle growth and development from the embryonic to the adult stage of an organism consists of a series of exquisitely regulated and orchestrated changes in expression of genes leading to muscle maturation. Genetic selection for body weight and muscle mass is ultimately based on differential expression of genes, and this approach has resulted in dramatic improvements in turkey growth rate, breast muscle mass, and feed efficiency. However, selection for these performance traits has inadvertently led to other undesirable traits including myopathies [26,27], cardiomyopathies [28,29], skeletal deficiencies [11,30], and meat quality defects such as the pale, soft, and exudative (PSE) condition [31,32] and associated decreased meat protein functionality [33]. The goals in the current study were to exploit the capability of a microarray approach to identify functional pathways of genes and thus characterize molecular events associated with muscle development and between genetic lines of turkeys with different growth rates.

Obvious phenotypic differences exist between RBC2 birds and those from F or commercial lines. The F line turkeys gain body weight faster and yield significantly heavier breast muscles than the RBC2 birds [12]. In addition, satellite cells from the F line have faster proliferation and differentiation rates than those from the RBC2 line [13]. Further, muscle damage with age is significant between lines as birds from the F line display a greater degree of muscle fiber fragmentation and areas of hypercontraction compared to those of the RBC2 [26]. These phenotypic differences are likely the results of differential gene expression throughout development.

This study identified over 3000 genes affected by developmental stage (Experiment 1), but only 16 genes were identified as being differentially expressed by overall effect of genetic line (Experiment 2). When considering effect of line within each developmental stage, the highest number of differences occurred early in development (18de stage). Expression changes in myogenic regulatory factor genes have previously been observed between the F and RBC2 lines at the embryonic stage [14], and satellite cells from F birds have been shown to have increased proliferation rates relative to RBC2 birds *in vitro *[13]. These results agree with the microarray observations and suggest that phenotypic differences between the two lines may largely be determined embryonically during myogenesis. It is possible that subtle differences in gene expression observed in the current study may be responsible for large phenotypic differences in body and breast muscle weight [12], breast muscle morphology [26], and meat quality characteristics [33] between the RBC2 and F lines at market age.

The Ingenuity Pathway Analysis (IPA) enabled the clustering of differentially expressed genes into functional categories, some of which were expected, while others were novel. Expected differences provided biological validation of the microarray approach, while the novel differences provided new paths to pursue in furthering our understanding of differences in turkey skeletal muscle growth and development. Many genes that may act as master regulators during development appeared in more than one Network, Function, or Canonical Pathway. The identified gene expression differences were grouped by function and discussed, highlighting several individual genes of interest within each group.

### Extracellular Matrix Genes

Previous studies have demonstrated that the ECM comprising the connective tissue surrounding muscle can interact with growth factors, regulate cellular signal transduction pathways, and affect growing and developing muscle fibers [34-36]. Genes that fell into numerous functions or canonical pathways as well as some genes that were not included in IPA networks were selected for this group. The ECM is composed mainly of collagens and non-fibrous glycoproteins like proteoglycans. The expression of three proteoglycans: *versican, glypican-1*, and *syndecan-4*, was quantified in this study. Membrane-associated proteoglycans in the ECM are known to regulate numerous growth factors, the major constituents that regulate activation, proliferation, and differentiation of satellite cells [8]. A predominant example is FGF2, a potent stimulator of muscle fiber proliferation and a strong inhibitor of differentiation [35,37]. This function would explain the high *FGF2 *expression at 18de and 1d in the current study, as these are periods of myoblast or satellite cell proliferation. The interaction between FGF2 and the heparan sulfate proteoglycans glypican-1 and syndecan-4 is required for high-affinity binding of FGF2 to its cellular receptor [35]. Differences in expression of *syndecan-4 *have been observed *in vitro *between developing RBC2 and F line satellite cells [15], and RBC2 satellite cell expression of *syndecan-4 *as well as *glypican-1 *were altered with the addition of FGF2 [38]. Satellite cells from *syndecan-4 *knockout mice display defective patterns of activation, proliferation, and differentiation [39], indicating a crucial role in muscle development that is also supported for turkeys in the current study. In addition, *syndecan-4 *and *FGF2 *appear to have similar expression profiles, especially at 1d. Versican is a chondroitin sulfate proteoglycan that was expressed predominantly at the 18de stage in this study. Fernandez et al. (1991) hypothesized that this protein's high embryonic expression at the time of fiber formation may be involved in fiber spacing, establishing the morphological structure of the muscle in chicks [40]. The space between muscle fibers may play a critical role in the water-holding capacity of mature muscle and may therefore be associated with quality defects like PSE meat. It is therefore possible that characteristics that contribute to meat quality are decided very early in myogenesis.

Dominant negative mutations in the *COL6A1 *gene, the only collagen that was investigated in the current study, are known to lead to Bethlem myopathy [41] and Ulrich congenital muscle dystrophy [42]; *COL6A1 *knockout mice exhibit sarcolemmal disorganization [43]. Taken together, it is clear that expression of genes whose proteins are located in the ECM of skeletal muscle likely contribute significantly to myogenesis in RBC2 and F turkeys.

### Cell Death/Apoptosis Genes

Cell death and apoptosis are necessary events in muscle differentiation and maintenance. In addition, other reports have shown similarities between mechanisms of apoptosis and myoblast differentiation, suggesting that similar pathways and effectors may be in use [44,45]. Therefore, it was logical to further evaluate the expression of several differentially expressed genes associated with these functions. For example, caspase-3, an "executioner" cysteine protease in apoptosis [46], is necessary for proper differentiation of C2C12 myoblasts [47]. However, several reports have shown that this role is separate from apoptosis [44,45] although it employs similar mechanisms, such as DNA strand breakage [48].

A component of the microfilament, GAS2, is a substrate for caspase-3 and is involved in microfilament reorganization, possibly an early event in apoptosis [49]. The protein is expressed in embryonic mouse limbs and involved in apoptosis during interdigital development; GAS2 may also be involved in myogenesis, possibly in the fusion of myoblasts to form myotubules [50]. In addition, GAS2 can regulate p53 action and apoptosis through its inhibition of calpain [51]. Nestin is the initial intermediate filament protein expressed during myogenesis [52-54], and phosphorylation of this protein is associated with the disassembly of intermediate filaments during mitosis [53]. Nestin is also associated with increased survival of rat vascular smooth muscle cells under stress [55]. Our results are in agreement as *nestin *is expressed predominantly at the 18de stage in turkey skeletal muscle when myoblasts are proliferating.

Another intriguing gene was *DAP*, which was expressed most highly at the 18de stage and decreased throughout development for both RBC2 and F birds. The human homologue of the gene was first identified in surviving HeLa cells treated with an antisense cDNA library and the cytokine interferon-γ to induce apoptosis [56]. Very little is known about this gene other than it seemed to be a candidate for positive mediators of cell death [56,57] and that it may regulate autophagy in injured planarians [58] and in amino acid-starved HeLa cells [59]. In addition, DAP may play a role as a regulator of turkey skeletal muscle proliferation and differentiation as shown by our group's recent work with satellite cells *in vitro *(Vellemen et al., manuscript in preparation).

### Ca^2+ ^Signaling/Muscle Function Genes

The Ca^2+ ^ion acts as a second messenger in cell signaling and in skeletal muscle contraction. Aberrant Ca^2+ ^signaling postmortem likely plays an important role in the occurrence of meat quality defects that can lead to significant economic losses in the pork and poultry industries. During the postmortem conversion of muscle to meat, increased Ca^2+ ^release from the sarcoplasmic reticulum while the muscle temperature is still high is thought to be responsible for skeletal muscle hypermetabolism that accelerates pH decline, leading to protein denaturation and loss of protein functionality [60,61]. A point mutation in the "halothane gene" or *ryanodine receptor 1 (RYR1)*, the skeletal muscle sarcoplasmic reticulum Ca^2+ ^release channel, has been identified in the pig [62] and has been associated with porcine stress syndrome (PSS), a sometimes fatal condition of malignant hyperthermia that is triggered by stress and is a major contributor to the development of PSE pork [60]. Genetic selection by pig breeders away from this mutation has not fully resolved the PSE problem. However, *RYR1 *became a logical candidate for examination in the turkey, a species that has been similarly selected for rapid lean muscle growth and also exhibits meat quality defects such as PSE. Further investigation of this protein in the turkey revealed a higher affinity for ryanodine in heavier commercial turkeys than those from the RBC2 line [63], suggesting a higher open-state probability of the Ca^2+ ^release channel. Multiple alternatively spliced products were later discovered in the avian skeletal muscle ryanodine receptor isoform, *αRYR*, in RBC2 and commercial turkeys [64], and these alternative splicing sites are clustered in regions of the gene associated with increased frequency of mutations [65]. Recently, alternative splicing and changes in *αRYR *expression have also been implicated in the occurrence of PSE meat in broiler chickens [66].

Several genes involved in Ca^2+ ^metabolism were identified as differentially expressed during development in this study. Calreticulin functions as a Ca^2+ ^-binding chaperone and aids in maintaining Ca^2+ ^homeostasis in the ER lumen of cells [67]. This protein also appears to be mainly expressed early in murine cardiac development and is essential for proper cardiac development [68]. Calmodulin is another Ca^2+ ^-binding protein that sensitizes RYR1 to activation at nanomolar concentrations of Ca^2+ ^and inhibits RYR1 at micromolar concentrations [69]. However, more recent work suggests that calmodulin is not an essential regulator of RYR1 [70]. Nevertheless, calmodulin plays a central role in calcium signal transduction and the developmentally related changes in gene expression suggest a key role for this protein.

Myostatin is a highly-conserved transforming growth factor-β (TGF-β) family member that strongly inhibits both hyperplasia and hypertrophy. Mutations or knockout of the gene result in an extreme increase in skeletal muscle mass in cattle and in mice [71]. In the current study, a dramatic decrease of *myostatin *expression was observed at 1d post-hatch, which agrees with our understanding of myostatin function as myofibers are rapidly undergoing hypertrophy at this stage. In addition, line differences at 18de and 16wk were observed, with RBC2 birds expressing *myostatin *significantly higher than F birds at 18de. This result could imply that F birds are able to undergo a higher degree of hyperplasia and actually produce more mature muscle fibers, leading to greater muscle mass. At 16wk, F-line birds expressed significantly more *myostatin*, suggesting that the faster growth rate of the heavier birds plateaus sooner than the slower-growing RBC2-line birds. Activin receptor type IIB (ActIIBR) functions as a serine/threonine kinase receptor for myostatin as well as other TGF- β family members, and binding of these ligands to ActIIBR activates the Smad signal transduction pathway to regulate gene expression [72]. Inhibition of myostatin activity by construction of a dominant-negative ActRIIB lacking a kinase domain resulted in increased muscle hyperplasia and hypertrophy in mice, as well as competition with the TGF-β family member follistatin [71]. Treatment of chicken fetal myoblasts with myostatin altered expression of genes involved in myogenic differentiation, cell architecture, energy metabolism, signal transduction and apoptosis [73], suggesting that increased *myostatin *expression in the current study could lead to similar changes.

Troponins are key proteins that are regulated by changes in intracellular Ca^2+ ^concentrations and are responsible for striated muscle contraction. The three subunits of troponin interact with each other to bind Ca^2+ ^(TNNC), inhibit myosin ATPase activity (TNNI), and to bind tropomyosin (TNNT) [74]. Transcript abundance of the fast/skeletal muscle isoform of TNNT, *TNNT3*, increased with development in the current study, peaking at 16wk. Interestingly, the cardiac isoform, *TNNT2*, was chiefly expressed in the embryonic skeletal muscle, and its expression decreased during skeletal muscle development, which is consistent with previous findings in chicken [75,76]. Expression of another slow-twitch muscle/cardiac isoform of troponin, *TNNI1*, followed this pattern as well. The expression of all of the *troponin *isoforms is also in agreement with what is known about myofibrillar protein expression in rat skeletal muscle, as slow/cardiac forms are expressed during the development of myofibrils with a switch to fast/skeletal forms during postnatal growth and muscle regeneration [77]. These genes involved in muscle growth and Ca^2+ ^signaling may be valuable candidates for changes observed in turkey skeletal muscle development.

### Miscellaneous/Genes of Unknown Function

One of the goals of the current study was to identify novel genes with unknown or uncertain functions that may play important roles in skeletal muscle development. These genes may play important roles in myogenesis and development even though their annotations and functions have yet to be defined or may not have clear roles in skeletal muscle. In the current study, one gene without known annotation but identified as very highly differentially expressed by microarray analysis was chosen for further confirmation by qPCR along with others that did not easily fit into a functional category. These genes all showed line differences at 1d, indicating a possible important role during muscle hypertrophy that may help explain phenotypic differences between the slow-growing randombred line and the line selected for increased 16-wk body weight.

Several genes identified in this study were first discovered and their activities characterized in tissue types other than skeletal muscle. For example, *spondin 2*, (also known as *mindin)*, which fell into the ECM group in our study, is expressed in spleen, lymph nodes, and dendritic cells and may be crucial to immune function [78]. In the current study, *spondin 2 *was expressed at highest levels at 1d post-hatch, over 11 times higher than its 18de expression in both turkey lines. The *MGP *gene was first cloned in chickens and found in highest levels in bones and relatively low levels in skeletal muscle [79]. Previous studies indicate its role in the inhibition of vascular calcification, as *MGP *knockout mice die within 2 months due to blood vessel rupture [80]. It may also be important in intracellular calcium homeostasis [81]. While *MGP *was grouped into the "Cell Death" function by the IPA software, its association with apoptosis is not clear [81]. It was another gene that appeared to be "turned on" at 1d post-hatch, with 80.28 and 55.38 times the 18de expression in RBC2 and F lines, respectively. Thus, this study has begun to uncover novel roles of these genes, which have been characterized in other tissues, in muscle growth and development.

Other interesting findings include the variation in the timing and extent of expression during development. A few stand-out genes were highly expressed at only one stage of development, clearly turned on or off in what appears to be carefully choreographed regulation. During the embryonic stage, when muscle cell hyperplasia is occurring, *versican, glypican-1, betaparvin, nestin, TNNI1*, and *TNNT2 *were expressed at least 5 times, and in the case of *nestin *greater than 100 times, higher than later developmental stages. *Spondin 2 *and *MGP *were both highly expressed at 1d posthatch with very little expression at 18de or 16wk. The *NT5C3 *and *TNNT3 *genes peaked at 16wk of development.

## Conclusions

Taken together, results from the current study demonstrated obvious differences in gene expression between three key developmental stages of turkey skeletal muscle growth. Although differences between the two genetic lines may be more subtle, the TSKMLO microarray platform proved useful in identifying candidate genes responsible for differences in muscle growth and opened the door for future experiments that focus on these candidate genes, their proteins, and their functions in turkey skeletal muscle.

## Authors' contributions

KRBS carried out all microarray and qPCR procedures, annotation and pathway analysis, and wrote the manuscript. RJT was involved in experimental design and statistically analyzed all microarray data. CWE, KMR, SGV, and GMS were involved in experimental design and drafting the manuscript. All authors read and approved the final manuscript.

## Supplementary Material

Additional File 1 **Supplementary Figure 1 (Figure S1)**. Experimental designs of microarray experiments. Click here for file

Additional File 2 **Supplementary Table 1 (Table S1)**. Top thirty down-regulated and up-regulated genes in Experiment 1 in each of the following comparisons: RBC2 18de:1d, RBC2 1d: 16wk, F 18de: 1d, F 1d:16wk, with fold changes, GenBank accession numbers, and putative annotations. Click here for file

Additional File 3 **Supplementary Table 2 (Table S2)**. Top down-regulated and up-regulated genes (RBC2:F) in Experiment 2 for the developmental stages 18de, 1d, and 16wk with fold changes, GenBank accession numbers, and putative annotations. Click here for file
