# Supplementary material for: Transcriptional profiling identifies differentially expressed genes in developing turkey skeletal muscle
Source: BMC Genomics. 2011 Mar 8;12:143. doi: 10.1186/1471-2164-12-143 (PMC3060885; doi:10.1186/1471-2164-12-143)
Supplement: Additional File 1 — Supplementary Figure 1 (Figure S1). Experimental designs of microarray experiments. [file 1471-2164-12-143-S1.PDF]

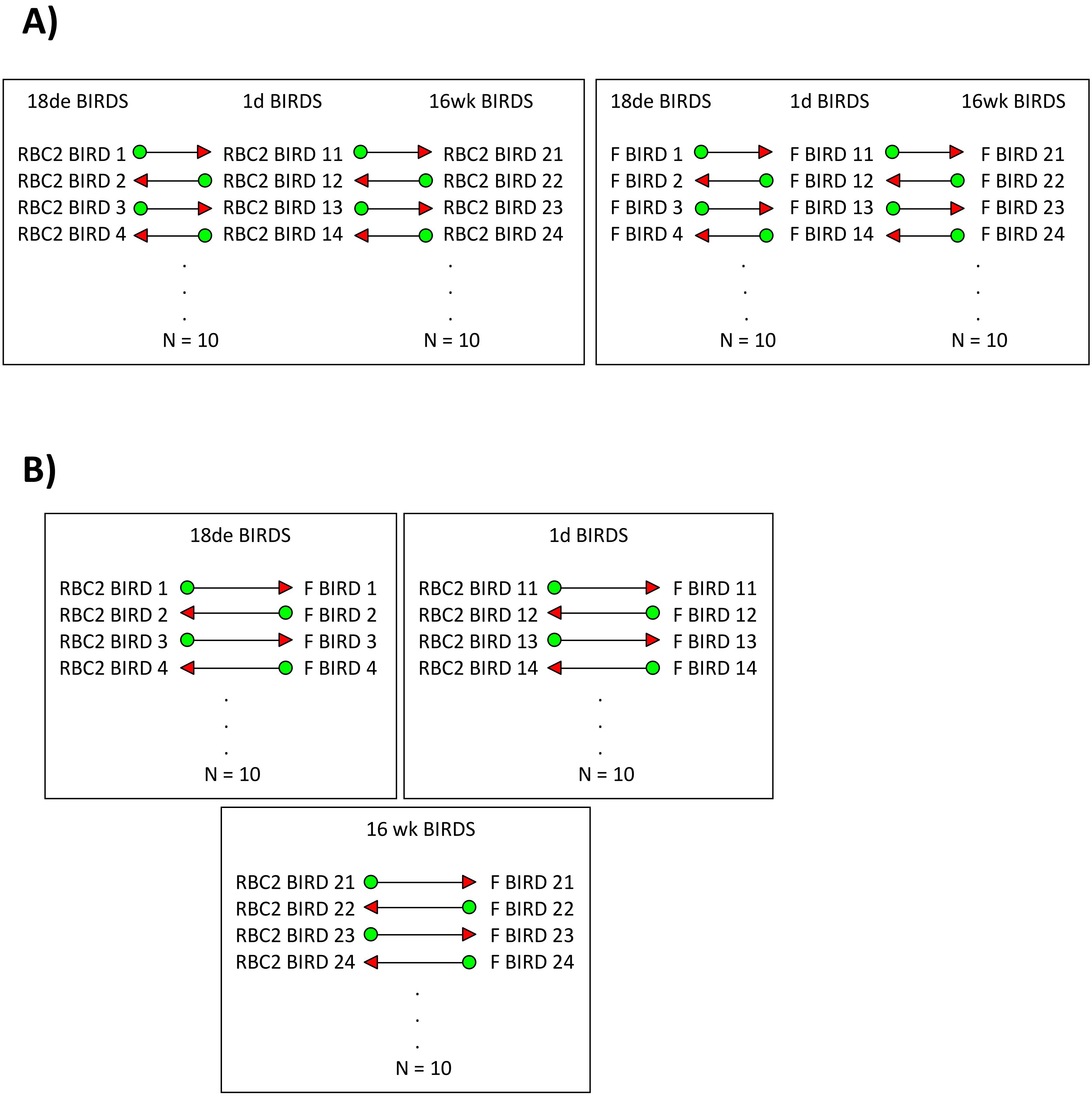
A)
18de BIRDS
1d BIRDS
16wk BIRDS
RBC2 BIRD 1
RBC2 BIRD 2
RBC2 BIRD 3
RBC2 BIRD 4
RBC2 BIRD 11
RBC2 BIRD 12
RBC2 BIRD 13
RBC2 BIRD 14
RBC2 BIRD 21
RBC2 BIRD 22
RBC2 BIRD 23
RBC2 BIRD 24
N = 10
N = 10
18de BIRDS
1d BIRDS
16wk BIRDS
F BIRD 1
F BIRD 2
F BIRD 3
F BIRD 4
F BIRD 11
F BIRD 12
F BIRD 13
F BIRD 14
F BIRD 21
F BIRD 22
F BIRD 23
F BIRD 24
N = 10
N = 10
B)
18de BIRDS
RBC2 BIRD 1
RBC2 BIRD 2
RBC2 BIRD 3
RBC2 BIRD 4
F BIRD 1
F BIRD 2
F BIRD 3
F BIRD 4
N = 10
1d BIRDS
RBC2 BIRD 11
RBC2 BIRD 12
RBC2 BIRD 13
RBC2 BIRD 14
F BIRD 11
F BIRD 12
F BIRD 13
F BIRD 14
N = 10
16 wk BIRDS
RBC2 BIRD 21
RBC2 BIRD 22
RBC2 BIRD 23
RBC2 BIRD 24
F BIRD 21
F BIRD 22
F BIRD 23
F BIRD 24
N = 10

Supplementary Figure 1. Experimental designs of microarray experiments. Each arrow represents one array; the arrow tails represent the sample labeled with Cy3 (green) fluorescent dye, while the arrow heads represent the sample labeled with Cy5 (red) fluorescent dye. RBC2 = randombred control turkey line; F = turkey line selected for increased 16wk weight. 18de (numbered 1-10 for each line) = 18d embryo (representative of muscle hyperplasia); 1d (numbered 11-20 for each line) = 1 day posthatch chick (representative of muscle hypertrophy); 16wk (numbered 21-30) = 16 week market weight bird. A) Experiment 1 contained a total of 40 arrays, comparing developmental stages (18de vs 1d and 1d vs 16wk) within each genetic line (RBC2 or F). B) Experiment 2 contained a total of 30 arrays, directly comparing genetic line (RBC2 vs F) for each developmental stage (18de or 1d or 16wk).
