# Supplementary material for: Transcriptional profiling identifies differentially expressed genes in developing turkey skeletal muscle
Source: BMC Genomics. 2011 Mar 8;12:143. doi: 10.1186/1471-2164-12-143 (PMC3060885; doi:10.1186/1471-2164-12-143)
Supplement: Additional File 2 — Supplementary Table 1 (Table S1). Top thirty down-regulated and up-regulated genes in Experiment 1 in each of the following comparisons: RBC2 18de:1d, RBC2 1d: 16wk, F 18de: 1d, F 1d:16wk, with fold changes, GenBank accession numbers, and putative annotations. [file 1471-2164-12-143-S2.PDF]

Supplementary Table 1. Top thirty down-regulated and up-regulated genes in Experiment 1 in each of the following comparisons: RBC2 18de:1d, RBC2 1d: 16wk, F 18de: 1d, F 1d:16wk, with fold changes, GenBank accession numbers, and putative annotations.

Array ID	Fold change RBC2 18de:1d	GenBank Accession #	Putative Annotation	FDR
Q1_Reed_1DPH_02_03_E13_197	-30.71	XM_425039	PREDICTED: Gallus gallus fructose-1,6-bisphosphatase 2 (FBP2)	5.84E-10
Q4_Reed_1DPH_cDNA_11_N04_062	-26.04	No significant similarity found	Unknown	1.34E-11
Contig[0303]	-24.90	NM_204405.1	Gallus gallus ankyrin repeat domain 1 (cardiac muscle) (ANKRD1)	1.24E-07
Q1_Reed_1DPH_cDNA_04_O19_303	-23.91	No significant similarity found	Unknown	1.10E-09
Contig[2858]	-19.36	XM_426680	PREDICTED: Gallus gallus hypothetical LOC429124 (LOC429124)	6.39E-13
Q1_Reed_16wkturkeyMuscle_02_E23	-18.89	NM_204405.1	Gallus gallus ankyrin repeat domain 1 (cardiac muscle) (ANKRD1)	2.29E-08
Q3_Reed_1DPH_cDNA_07_P09_144	-18.66	NM_001006443	Gallus gallus solute carrier family 25 (mitochondrial carrier; adenine nucleotide translocator), member 4 (SLC25A4), nuclear gene encoding mitochondrial protein	0
Contig[0902]	-15.10	NM_205525	Gallus gallus apolipoprotein A-I (APOA1)	0
Q4_Reed_16wkturkeymuscle_10_H04	-14.08	No significant similarity found	Unknown	2.81E-11
Q2_Reed_16wkturkeymuscle_08_M18	-13.58	No significant similarity found	Unknown	2.45E-12
Contig[2711]	-13.50	XM_425039	PREDICTED: Gallus gallus fructose-1,6-bisphosphatase 2 (FBP2)	1.29E-09
Contig[2553]	-13.48	NM_205044	Gallus gallus matrix Gla protein (MGP)	0
Q4_Reed_16wkTurkeyMuscle_03_F12	-11.95	NM_001006443	Gallus gallus solute carrier family 25 (mitochondrial carrier; adenine nucleotide translocator), member 4 (SLC25A4), nuclear gene encoding mitochondrial protein	1.79E-12
Contig[2639]	-11.86	NM_204629	Gallus gallus aldo-keto reductase family 1, member B10 (aldose reductase) (AKR1B10)	2.24E-11

Q4_Reed_16wkTurkeyMuscle_02_N10	-11.57	BX663531	Chicken DNA sequence from clone WAG-18M1, complete sequence	2.83E-10
Contig[0922]	-11.57	NM_204922	Gallus gallus troponin T type 3 (skeletal, fast) (TNNT3)	1.99E-11
Contig[1575]	-11.39	AJ719983	Gallus gallus mRNA for hypothetical protein, clone 9b10	7.14E-09
Contig[1412]	-11.08	AF226597	Meleagris gallopavo fast skeletal muscle troponin T isoform 1e17	1.20E-08
Q1_Reed_1DPH_cDNA_08_K13_203	-11.05	AF226597	Meleagris gallopavo fast skeletal muscle troponin T isoform 1e17 mRNA	8.06E-11
Q1_Reed_16wkturkeymuscle_09_G17	-10.86	XM_424899	PREDICTED: Gallus gallus creatine kinase, mitochondrial 2 (sarcomeric) (CKMT2)	6.09E-10
HSP27-C	-10.64	NM_205290	Gallus gallus heat shock 27kDa protein 1 (HSPB1)	1.45E-10
Q4_Reed_16wkturkeymuscle_10_H04-C	-9.64	No significant similarity found	Unknown	2.49E-11
Q1_Reed_1DPH_cDNA_06_O15_239	-9.47	AF226597	Meleagris gallopavo fast skeletal muscle troponin T isoform 1e17	3.75E-09
Q4_Reed_16wkTurkeyMuscle_03_F12-C	-9.19	NM_001006443	Gallus gallus solute carrier family 25 (mitochondrial carrier; adenine nucleotide translocator), member 4 (SLC25A4), nuclear gene encoding mitochondrial protein	8.92E-11
Contig[1199]	-9.15	NM_205284	Gallus gallus lactate dehydrogenase A (LDHA)	1.74E-05
Q3_Reed_16wkTurkeyMuscle_04_B23	-9.08	AF210256	Meleagris gallopavo fast skeletal muscle troponin T	2.39E-08
Q2_Reed_1DPH_cDNA_02_G02_023	-9.01	XM_418671	PREDICTED: Gallus gallus pyruvate dehydrogenase kinase, isozyme 4 (PDK4)	2.05E-04
Contig[0075]	-8.69	NM_205119	Gallus gallus enolase (LOC396016)	7.56E-10
Q1_Reed_1DPH_cDNA_05_I19_297	-8.59	AJ719983	Gallus gallus mRNA for hypothetical protein, clone 9b10	6.161E-10
Contig[0809]	-8.56	M22154	Chicken skeletal muscle troponin T variant Tnt-1	2.62E-09
Contig[1084]-C	13.87	XM_414685	PREDICTED: Gallus gallus similar to betaine homocysteine methyl transferase (LOC416371)	8.50E-10
Q4_Reed_18d_tembryo_03_N24_382	12.72	XM_001235103	PREDICTED: Gallus gallus hypothetical protein LOC771886	3.85E-11

Contig[0001]	10.48	XM_421512	PREDICTED: Gallus gallus methionine adenosyltransferase I, alpha (MAT1A)	1.64E-10
Contig[2857]	10.43	NM_205009	Gallus gallus keratin 19 (KRT19)	1.85E-09
Contig[0574]	10.36	NM_205449	Gallus gallus troponin T type 2 (cardiac) (TNNT2)	9.79E-09
Contig[beta-H_globin_(LOC428114	10.02	NM_001031489	Gallus gallus hemoglobin, epsilon 1 (HBE1)	3.64E-10
Contig[2640]	9.64	XM_414990	PREDICTED: Gallus gallus tubulin, alpha 3 (TUBA3)	4.88E-09
Q4_Reed_um_mg_cDNA1_04_92D12	8.97	XM_424523	PREDICTED: Gallus gallus similar to Feather keratin I (Keratin gene C protein) (F-ker) (LOC426914)	8.06E-11
Contig[cardiac_troponin_T_isofo	8.91	M10013	Chicken cardiac troponin T form I	8.07E-09
Contig[0566]	8.90	U59287	Gallus gallus paranemin	4.47E-12
Contig[0002]	8.72	XM_414685	PREDICTED: Gallus gallus similar to betaine homocysteine methyl transferase (LOC416371)	2.94E-08
Q1_Reed_UM_Mg_cDNA1_05_78F10	8.33	XM_420586	PREDICTED: Gallus gallus protein phosphatase 1, regulatory (inhibitor) subunit 3B (PPP1R3B)	3.26E-10
Q2_Reed_18d_tembryo_02_G14_215	8.26	XM_420800	PREDICTED: Gallus gallus hypothetical LOC422854 (LOC422854)	2.26E-12
Contig[troponin_C_type_1_(slow)-C	8.19	NM_205133	Gallus gallus troponin C type 1 (slow) (TNNC1)	1.11E-11
Q3_Reed_1DPH_cDNA_01_N15_238	8.14	No significant similarity found	Unknown	1.70E-13
Q1_Reed_UM_Mg_cDNA1_05_53E07	7.60	XM_416567	PREDICTED: Gallus gallus similar to uroplakin 1B; tetraspan (LOC418345)	1.59E-10
Contig[0541]	7.20	U37118	Coturnix coturnix troponin I slow skeletal muscle isoform	9.81E-10
Contig[1084]	7.12	XM_414685	PREDICTED: Gallus gallus similar to betaine homocysteine methyl transferase (LOC416371)	1.19E-07
Contig[myosin__light_chain_2__r	6.95	XM_415166	PREDICTED: Gallus gallus myosin, light chain 2, regulatory, cardiac, slow (MYL2)	7.76E-10
Q3_Reed_16wkTurkeyMuscle_04_P01	6.85	No significant similarity found	Unknown	9.17E-09

Q4_Reed_UM_Mg_cDNA1_10_60D08	6.83	XM_416866	PREDICTED: Gallus gallus similar to PP2A B subunit PR48 (LOC418670)	6.39E-13
Q2_Reed_um_Mg_cDNA1_09_18B03	6.79	XM_416315	PREDICTED: Gallus gallus hypothetical LOC418079 (LOC418079)	1.17E-11
Contig[1121]	6.78	XM_421512	PREDICTED: Gallus gallus methionine adenosyltransferase I, alpha (MAT1A)	2.84E-09
Contig[1017]	6.65	XM_414990	PREDICTED: Gallus gallus tubulin, alpha 3 (TUBA3)	2.28E-08
Contig[0417]	6.57	NM_001079481	Gallus gallus actin, alpha, cardiac muscle 1 (ACTC1)	1.97E-06
Q2_Reed_1DPH_cDNA_04_E24_373-C	6.56	AY560111	Meleagris gallopavo myogenin	1.95E-08
Contig[cardiac_troponin_T__comp	6.56	NM_205449	Gallus gallus troponin T type 2 (cardiac) (TNNT2)	3.02E-07
Contig[0064]	6.52	NM_001013397	Gallus gallus myosin, heavy chain 6, cardiac muscle, alpha (MYH6)	1.30E-06
Q1_Reed_um_Mg_cDNA_01_57A08	6.51	AY005139.1	Meleagris gallopavo cardiac troponin T isoform	7.14E-09
Q4_Reed_um_Mg_cDNA1_09_71G09	6.43	XM_414685	PREDICTED: Gallus gallus similar to betaine homocysteine methyl transferase (LOC416371)	1.48E-06
Q3_Reed_UM_Mg_cDNA1_07_62F08	6.26	XM_422072	PREDICTED: Gallus gallus similar to Ku (p70/p80) protein (LOC424222)	0
Q2_Reed_um_Mg_cDNA1_08_81A11-C	6.00	XM_421512	PREDICTED: Gallus gallus methionine adenosyltransferase I, alpha (MAT1A)	3.51E-08
Q2_Reed_um_Mg_cDNA_01_27C04	6.00	XM_416866	PREDICTED: Gallus gallus similar to PP2A B subunit PR48 (LOC418670)	1.70E-13
Q1_Reed_um_mg_cDNA1_04_28D04	5.97	AC190152	Gallus gallus BAC clone CH261-171P12 from chromosome z	1.09E-08
Proteoglycan	5.67	NM_204787	Gallus gallus versican (VCAN)	1.19E-08

**Array ID**	**Fold change RBC2 1d:16wk**	**GenBank Accession #**	**Putative Annotation**	**FDR**
Contig[1485]	-42.98	XM_001236934	PREDICTED: Gallus gallus similar to Chain A, The Crystal StructureOf Human Barrier-To-Autointegration Factor (Baf) (LOC777489)	1.21E-07

Contig[0254]	-24.76	NM_205348.1	Gallus gallus collagen, type VI, alpha 2 (COL6A2)	5.29E-13
Q4_Reed_UM_Mg_cDNA1_10_85E11	-22.09	XM_423897.2	PREDICTED: Gallus gallus semaphorin 3B (SEMA3B)	0
Contig[beta_globulin__complete_	-16.60	AY775302	Gallus gallus beta globin protein 1 (BGP1)	3.87E-10
Contig[0414]-distance-3	-15.67	NM_204289	Gallus gallus heat shock protein 90kDa beta (Grp94), member 1 (HSP90B1)	7.77E-11
Contig[1673]	-12.20	NM_205096	Gallus gallus high density lipoprotein binding protein (vigilin) (HDLBP)	1.08E-11
Contig[0012]-mismatch-5	-11.21	NM_001031405	Gallus gallus coatomer protein complex, subunit alpha (COPA)	9.26E-08
Contig[0543]	-11.14	AF540410.1	Meleagris gallopavo microsatellite MNT-107	2.03E-12
Contig[2558]	-10.60	XM_419634	PREDICTED: Gallus gallus similar to homeodomain interacting protein kinase 3 (LOC421597)	4.11E-06
Contig[0436]	-10.10	NM_001079714	Gallus gallus collagen, type I, alpha 2 (COL1A2)	2.53E-08
Q4_Reed_um_mg_cDNA1_02_20D03	-9.67	XM_001231966	PREDICTED: Gallus gallus similar to Cks1 protein homologue (LOC768830)	3.11E-10
a-RYR:1245-1437:j2	-9.64	EU177005	Meleagris gallopavo skeletal muscle ryanodine receptor isoform alpha (alpha-RYR), alternatively spliced	8.31E-11
Q3_Reed_um_mg_cDNA1_03_42B06	-9.41	X57998.1	G.domesticus gene for collagen alpha 1 type VI	1.76E-08
Contig[0969]	-9.39	XM_418088	PREDICTED: Gallus gallus N-myristoyltransferase 1 (NMT1)	4.54E-09
Activin_IIB_Receptor-mismatch-5	-8.62	NM_204317	Gallus gallus activin A receptor, type IIB (ACVR2B)	0
Contig[0075]	-8.43	NM_205119	Gallus gallus enolase (LOC396016)	6.82E-12
Contig[0191]	-8.17	XM_415774	PREDICTED: Gallus gallus similar to Unc-45 homolog B (C. elegans) (LOC417527)	2.80E-09
Q3_Reed_16wkTurkeyMuscle_04_B05	-7.83	No significant similarity found	Unknown	3.94E-11
Activin_IIB_Receptor-mismatch-3	-7.72	NM_204317	Gallus gallus activin A receptor, type IIB (ACVR2B)	0

Q4_Reed_um_Mg_cDNA1_09_84D11	-7.57	No significant similarity found	Unknown	2.69E-11
Q1_Reed_16wkturkeyMuscle_01_E07	-7.42	No significant similarity found	Unknown	1.32E-08
Contig[2843]	-7.29	XM_414471	PREDICTED: Gallus gallus CXXC finger 5 (CXXC5)	9.27E-11
cMet-distance-m	-7.12	NM_205212	Gallus gallus met proto-oncogene (hepatocyte growth factor receptor) (MET)	1.57E-10
Activin_IIB_Receptor-distance-3	-7.09	NM_204317	Gallus gallus activin A receptor, type IIB (ACVR2B)	5.99E-12
Q1_Reed_1DPH_cDNA_12_A17_257	-6.82	NM_001031597	Gallus gallus poly(A) binding protein, cytoplasmic 1 (PABPC1)	1.01E-12
Contig[2731]	-6.81	CR353565	Gallus gallus finished cDNA, clone ChEST61a21	1.39E-08
Contig[2790]	-6.72	XM_414970	PREDICTED: Gallus gallus smoothened (LOC395949)	3.87E-10
Q4_Reed_18d_tembryo_06_J12_186	-6.62	NM_001012697	Gallus gallus membrane associated guanylate kinase, WW and PDZ domain containing 3 (MAGI3)	6.51E-09
Contig[0365]	-6.28	DQ217219	Taeniopygia guttata clone 0058P0014B06 putative calmodulin variant 1 mRNA	7.55E-08
Contig[0032]	-6.25	NM_205469	Gallus gallus pyruvate kinase, muscle (PKM2)	6.64E-07
Q1_Reed_1DPH_cDNA_02_I03_041	-5.99	XM_002194344	PREDICTED: Taeniopygia guttata zinc finger protein 644 (LOC100219692)	2.30E-10
Q3_Reed_1DPH_cDNA_03_N01_014	-5.97	CR338903	Gallus gallus finished cDNA, clone ChEST984e13	4.40E-10
Contig[0143]	-4.60	NM_205519.1	Gallus gallus ATPase, Ca++ transporting, cardiac muscle, fast twitch 1 (ATP2A1)	0
Q2_Reed_um_Mg_cDNA_01_22F03	42.37	CR386282.1	Gallus gallus finished cDNA, clone ChEST55p6	3.11E-13
Q1_Reed_1DPH_cDNA_05_I17_265	38.59	XM_414665.2	PREDICTED: Gallus gallus transcription elongation regulator 1 (TCERG1)	0
Q3_Reed_18d_tembryo_01_N01_014	30.42	NM_001030850.1	Gallus gallus amyloid beta (A4) precursor protein-binding, family A, member 2 binding protein (APBA2BP)	0
a-RYR:1245-1325:in	28.55	EU177005	Meleagris gallopavo skeletal muscle ryanodine receptor isoform alpha (alpha-RYR), alternatively spliced	0

Contig[0465]	27.35	NM_001030339	Gallus gallus CD81 molecule (CD81)	1.11E-11
Q3_Reed_18d_tembryo_05_F13_198	19.53	NM_204374.1	Gallus gallus serine/threonine kinase 25 (STE20 homolog, yeast) (STK25)	0
Q4_Reed_1DPH_cDNA_04_B18_274	18.10	NM_001039277.1	Gallus gallus signal recognition particle receptor ('docking protein') (SRPR)	0
Contig[0063]	17.17	XR_036513	PREDICTED: Ornithorhynchus anatinus similar to aldolase A, fructose-bisphosphate, (LOC100073366)	3.53E-10
Q3_Reed_um_mg_cDNA1_04_14F02	16.33	No significant similarity found	Unknown	1.51E-13
Q3_Reed_1DPH_cDNA_04_P15_240	15.32	XM_414311.1	PREDICTED: Gallus gallus similar to Hypothetical protein MGC76019 (LOC415968)	5.82E-13
Contig[0743]	15.09	NM_204152	Gallus gallus ribosomal protein L6 (RPL6)	5.49E-07
Q2_Reed_um_Mg_cDNA_01_43C06	14.88	XM_414227	PREDICTED: Gallus gallus hypothetical LOC415867 (LOC415867)	3.14E-10
Contig[0107]	14.45	XM_414580	PREDICTED: Gallus gallus similar to RIKEN cDNA 1810073G14 (LOC416262)	4.23E-10
Contig[0453]	13.31	AC192554	Gallus gallus BAC clone CH261-112M7 from chromosome z	4.33E-10
Contig[0003]	13.20	NM_205519	Gallus gallus ATPase, Ca++ transporting, cardiac muscle, fast twitch 1 (ATP2A1)	1.13E-07
Contig[1115]	13.15	XM_420681	PREDICTED: Gallus gallus similar to FSHD region gene 1 (LOC422726)	2.39E-10
Q4_Reed_1DPH_cDNA_01_N14_222	12.57	XM_001234562.1	PREDICTED: Gallus gallus similar to Potassium voltage-gated channel, Isk-related family, member 4 (LOC771271)	1.98E-13
Q1_Reed_1DPH_cDNA_02_G09_135	12.50	No significant similarity found	Unknown	1.33E-08
Akt2	12.46	AF181260	Gallus gallus protein serine/threonine kinase (AKT2)	0
Q2_Reed_18d_tembryo_06_O04_063	11.92	XM_415553	PREDICTED: Gallus gallus similar to NADPH-dependent FMN and FAD containing oxidoreductase (LOC417280)	7.02E-08
Contig[2175]	11.81	M59183	Chicken lysyl hydroxylase	1.13E-08
Contig[0822]	11.66	AY393845	Gallus gallus calreticulin	1.04E-09

Contig[0047]	11.34	NM_001097646	Gallus gallus family with sequence similarity 3, member C (FAM3C)	6.16E-10
Contig[0012]-distance-3	11.31	NM_001031405	Gallus gallus coatomer protein complex, subunit alpha (COPA)	7.12E-11
Contig[0811]	11.29	NM_204216	Gallus gallus damage-specific DNA binding protein 1, 127kDa (DDB1)	5.43E-09
Contig[0923]	11.26	XM_001232051	PREDICTED: Gallus gallus similar to Gu protein (LOC768898)	9.05E-09
Q1_Reed_UM_Mg_cDNA1_06_60D08	11.16	NM_204410.1	Gallus gallus secreted protein, acidic, cysteine-rich (osteonectin) (SPARC)	0
Q2_Reed_18d_tembryo_04_C22_339	11.09	NM_001030908	Gallus gallus Friend leukemia virus integration 1 (FLI1)	1.39E-09
Contig[0768]	11.01	NM_204496	Gallus gallus heterogeneous nuclear ribonucleoprotein H1 (H)(HNRNPH1)	1.39E-09
Contig[0006]	10.97	NM_204228	Gallus gallus myosin, heavy chain 2, skeletal muscle, adult (MYH2)	4.60E-10
Contig[0844]	10.87	U47273	Gallus gallus tissue transglutaminase	4.30E-09

**Array ID**	**Fold change F 18de:1d**	**GenBank Accession #**	**Putative Annotation**	**FDR**
Q4_Reed_1DPH_cDNA_11_N04_062	-22.17	No significant similarity found	Unknown	5.17E-11
Q1_Reed_1DPH_cDNA_04_O19_303	-21.43	XM_002196318	PREDICTED: Taeniopygia guttata similar to CCAAT/enhancer binding protein delta (LOC100222090)	4.34E-09
Q1_Reed_1DPH_02_03_E13_197	-17.23	XM_425039	PREDICTED: Gallus gallus fructose-1,6-bisphosphatase 2 (FBP2)	4.39E-08
Q3_Reed_1DPH_cDNA_07_P09_144	-16.58	NM_001006443	Gallus gallus solute carrier family 25 (mitochondrial carrier; adenine nucleotide translocator), member 4 (SLC25A4), nuclear gene encoding mitochondrial protein	0
Contig[0303]	-15.94	AB082934	Gallus gallus carp mRNA for cardiac ankyrin repeat protein	2.27E-06
Contig[0902]	-15.22	NM_205525	Gallus gallus apolipoprotein A-I (APOA1)	0

Contig[2553]	-12.72	NM_205044	Gallus gallus matrix Gla protein (MGP)	0
Contig[2858]	-11.74	XM_426680	PREDICTED: Gallus gallus hypothetical LOC429124	5.14E-11
Q1_Reed_16wkturkeyMuscle_02_E23	-11.58	AB082934	Gallus gallus carp mRNA for cardiac ankyrin repeat protein	9.25E-07
Contig[2639]	-11.33	NM_204629	Gallus gallus aldo-keto reductase family 1, member B10 (aldose reductase) (AKR1B10)	4.83E-11
Q4_Reed_16wkturkeymuscle_10_H04	-9.47	No significant similarity found	Unknown	1.41E-09
Contig[0592]	-9.40	XM_418053	PREDICTED: Gallus gallus BTG family, member 2 (BTG2)	4.11E-07
Q4_Reed_16wkTurkeyMuscle_03_F12	-9.31	NM_001006443	Gallus gallus solute carrier family 25 (mitochondrial carrier; adenine nucleotide translocator), member 4 (SLC25A4), nuclear gene encoding mitochondrial protein	2.94E-11
HSP27-C	-9.28	NM_205290	Gallus gallus heat shock 27kDa protein 1 (HSPB1)	8.71E-10
Q4_Reed_16wkTurkeyMuscle_03_F12-C	-8.69	NM_001006443	Gallus gallus solute carrier family 25 (mitochondrial carrier; adenine nucleotide translocator), member 4 (SLC25A4), nuclear gene encoding mitochondrial protein	2.71E-10
Q2_Reed_16wkturkeymuscle_08_M18	-8.59	No significant similarity found	Unknown	2.71E-10
Contig[1575]	-8.21	AJ719983	Gallus gallus mRNA for hypothetical protein, clone 9b10	1.78E-07
Contig[0908]	-8.08	M22156	Chicken skeletal muscle troponin T variant Tnt-3	1.04E-10
Q1_Reed_1DPH_cDNA_08_G23_359	-7.56	XM_420847	PREDICTED: Gallus gallus spondin 2, extracellular matrix protein (SPON2)	5.85E-13
Q1_Reed_1DPH_cDNA_05_I19_297	-7.35	AJ719983	Gallus gallus mRNA for hypothetical protein, clone 9b10	5.72E-09
HSP27	-7.28	NM_205290	Gallus gallus heat shock 27kDa protein 1 (HSPB1)	6.46E-09
Q4_Reed_16wkTurkeyMuscle_02_N10	-7.28	BX663531	Chicken DNA sequence from clone WAG-18M1	3.47E-08
Contig[0922]	-7.20	NM_204922	Gallus gallus troponin T type 3 (skeletal, fast) (TNNT3)	2.02E-09

Contig[0639]	-7.13	AB021180	Gallus gallus mRNA for skeletal myosin heavy chain	3.75E-08
Contig[2711]	-6.89	XM_425039	PREDICTED: Gallus gallus fructose-1,6-bisphosphatase 2 (FBP2)	7.84E-07
Q1_Reed_1DPH_cDNA_08_K13_203	-6.74	AF226597	Meleagris gallopavo fast skeletal muscle troponin T isoform 1e17	1.55E-08
Contig[1412]	-6.47	AF226597	Meleagris gallopavo fast skeletal muscle troponin T isoform 1e17	2.09E-06
Q3_Reed_16wkturkeymuscle_07_B15	-6.45	AF210256	Meleagris gallopavo fast skeletal muscle troponin T mRNA	4.67E-08
Q2_Reed_16wkturkeyMuscle_01_I04	-6.41	XM_002194494	PREDICTED: Taeniopygia guttata TSC22 domain family, member 3 (LOC100227519)	1.94E-09
Q2_Reed_16wkturkeymuscle_06_M04	-6.38	No significant similarity found	Unknown	2.37E-06
Q4_Reed_18d_tembryo_03_N24_382	13.86	XM_001235103	PREDICTED: Gallus gallus hypothetical protein LOC771886	3.09E-11
Contig[myosin__light_chain_2__r	12.12	XM_415166	PREDICTED: Gallus gallus myosin, light chain 2, regulatory, cardiac, slow (MYL2)	8.04E-12
Q4_Reed_um_mg_cDNA1_04_92D12	10.26	XM_424523	PREDICTED: Gallus gallus similar to Feather keratin I (Keratin gene C protein) (F-ker) (LOC426914)	4.49E-11
Contig[beta-H_globin_(LOC428114	10.14	NM_001031489	Gallus gallus hemoglobin, epsilon 1 (HBE1)	6.79E-10
Contig[0574]	9.69	NM_205449	Gallus gallus troponin T type 2 (cardiac) (TNNT2)	3.10E-08
Contig[troponin_C_type_1_(slow)-C	9.67	NM_205133	Gallus gallus troponin C type 1 (slow) (TNNC1)	3.57E-12
Contig[2857]	9.28	NM_205009	Gallus gallus keratin 19 (KRT19)	1.04E-08
Contig[cardiac_troponin_T_isofo	9.17	M10013	Chicken cardiac troponin T form I	1.12E-08
Q2_Reed_18d_tembryo_02_G14_215	8.61	XM_420800	PREDICTED: Gallus gallus hypothetical LOC422854	3.57E-12
Contig[0566]	8.49	U59287	Gallus gallus paranemin	1.26E-11
Contig[0001]	7.69	XM_421512	PREDICTED: Gallus gallus methionine adenosyltransferase I, alpha (MAT1A)	6.12E-09

Contig[troponin_C_type_1_(slow)	7.51	NM_205133	Gallus gallus troponin C type 1 (slow) (TNNC1)	0
Q1_Reed_UM_Mg_cDNA1_05_53E07	7.39	XM_416567	PREDICTED: Gallus gallus similar to uroplakin 1B; tetraspan (LOC418345)	3.88E-10
Contig[1084]-C	7.38	XM_414685	PREDICTED: Gallus gallus similar to betaine homocysteine methyl transferase (LOC416371)	3.39E-07
Q3_Reed_16wkTurkeyMuscle_04_P01	7.12	No significant similarity found	Unknown	1.12E-08
Q1_Reed_UM_Mg_cDNA1_05_78F10	6.77	BX929364	Gallus gallus finished cDNA, clone ChEST184n3	5.2E-09
Contig[cardiac_troponin_T__comp	6.69	NM_205449	Gallus gallus troponin T type 2 (cardiac) (TNNT2)	4.94E-07
Q2_Reed_um_Mg_cDNA1_09_18B03	6.55	XM_416315	PREDICTED: Gallus gallus hypothetical LOC418079	2.71E-11
Q1_Reed_um_Mg_cDNA_01_57A08	6.39	XM_420586	PREDICTED: Gallus gallus protein phosphatase 1, regulatory (inhibitor) subunit 3B (PPP1R3B)	1.53E-08
Q1_Reed_um_Mg_cDNA1_08_84D11	6.33	XM_418162	PREDICTED: Gallus gallus similar to Krt42 protein (LOC420039)	2.94E-11
Q2_Reed_1DPH_cDNA_04_E24_373-C	6.04	XM_420586	PREDICTED: Gallus gallus protein phosphatase 1, regulatory (inhibitor) subunit 3B (PPP1R3B)	7.86E-08
Myogenin	6.01	NM_204184	Gallus gallus myogenin (myogenic factor 4) (MYOG)	3.22E-08
Q1_Reed_um_mg_cDNA1_04_28D04	5.94	AC190152	Gallus gallus BAC clone CH261-171P12 from chromosome z	2.10E-08
Contig[0541]	5.92	U37118	Coturnix coturnix troponin I slow skeletal muscle isoform	1.53E-08
Contig[2640]	5.87	XM_414990	PREDICTED: Gallus gallus tubulin, alpha 3 (TUBA3)	8.40E-07
Q3_Reed_1DPH_cDNA_01_N15_238	5.83	No significant similarity found	Unknown	1.16E-11
Contig[0457]	5.75	NM_204155	Gallus gallus SET and MYND domain containing 1 (SMYD1)	4.89E-11
Q3_Reed_UM_Mg_cDNA1_06_47G06	5.61	AF374417	Meleagris gallopavo cardiac troponin T gene, exons 7 through 10	1.35E-08
Q3_Reed_18d_tembryo_07_D23_356	5.55	NM_001004376	Gallus gallus hemoglobin, alpha 1 (HBA1)	1.50E-05

Contig[alpha-globin__complete_C	5.55	AY016020	Gallus gallus alpha globin gene cluster	1.86E-05
Contig[beta_globulin__complete_	5.54	AY775302	Gallus gallus beta globin protein 1 (BGP1)	0.000134
Proteoglycan	5.54	NM_204787	Gallus gallus versican (VCAN)	2.79E-08
Contig[0002]	5.52	XM_414685	PREDICTED: Gallus gallus similar to betaine homocysteine methyl transferase (LOC416371)	3.44E-06
Contig[2261]	5.43	NM_205033	Gallus gallus nestin (NES)	4.92E-12

**Array ID**	**Fold change F 1d:16wk**	**GenBank Accession #**	**Putative Annotation**	**FDR**
Contig[2731]	-22.81	CR353565	Gallus gallus finished cDNA, clone ChEST61a21	1.60E-12
Contig[0639]	-21.24	AB021180	Gallus gallus mRNA for skeletal myosin heavy chain	2.59E-11
Q3_Reed_um_Mg_cDNA1_08_75C10	-17.73	NM_001007477	Gallus gallus parvalbumin (PVALB)	6.36E-14
Contig[0849]	-15.89	XM_420634	PREDICTED: Gallus gallus similar to calcineurin-binding protein calsarcin-1 (LOC422682)	2.31E-05
Q2_Reed_1DPH_cDNA_02_G02_023	-13.40	XM_418671	PREDICTED: Gallus gallus pyruvate dehydrogenase kinase, isozyme 4 (PDK4)	9.02E-12
Contig[2639]	-12.45	NM_204629	Gallus gallus aldo-keto reductase family 1, member B10 (aldose reductase) (AKR1B10)	5.32E-07
Q1_Reed_1DPH_cDNA_08_I13_201-C	-11.25	NM_204745	Gallus gallus glycine amidinotransferase (L-arginine:glycineamidinotransferase) (GATM), nuclear gene encoding mitochondrial protein	3.80E-11
Contig[cardiac_myosin_alkali_li	-10.56	X13863	Chicken cardiac myosin alkali light chain gene exon 1	3.34E-12
Q4_Reed_18d_tembryo_07_N22_350	-10.47	XM_420634	PREDICTED: Gallus gallus similar to calcineurin-binding protein calsarcin-1 (LOC422682)	1.69E-08
Q4_Reed_1DPH_cDNA_06_L20_316	-9.52	CR353565	Gallus gallus finished cDNA, clone ChEST61a21	3.33E-10
Contig[0808]	-9.40	J02714	Chicken embryonic myosin heavy chain gene	4.62E-08

Contig[0064]	-9.39	J02714	Chicken embryonic myosin heavy chain gene	4.59E-08
Q4_Reed_UM_Mg_cDNA1_10_44D06	-8.68	AC186822	Gallus gallus BAC clone CH261-160D4 from chromosome z	5.48E-07
Contig[0683]	-8.22	NM_204745	Gallus gallus glycine amidinotransferase (L-arginine:glycineamidinotransferase) (GATM), nuclear gene encoding mitochondrial protein	1.83E-05
Q2_Reed_1DPH_cDNA_07_M06_093	-8.12	XM_418671	PREDICTED: Gallus gallus pyruvate dehydrogenase kinase, isozyme 4 (PDK4)	0
Q1_Reed_UM_Mg_cDNA1_10_32H04	-7.94	XM_415995	PREDICTED: Gallus gallus leiomodin 2 (cardiac) (LMOD2)	6.36E-14
Q1_Reed_1DPH_cDNA_08_G23_359	-7.93	XM_420847	PREDICTED: Gallus gallus spondin 2, extracellular matrix protein (SPON2)	6.36E-14
Q1_Reed_1DPH_cDNA_11_K17_267	-7.35	XM_420847	PREDICTED: Gallus gallus spondin 2, extracellular matrix protein (SPON2)	1.21E-07
Contig[0438]-C	-7.09	J02714	Chicken embryonic myosin heavy chain gene	6.19E-11
Q3_Reed_1DPH_cDNA_10_N21_334	-6.79	NM_206985	Gallus gallus tenomodulin (TNMD)	1.95E-11
Contig[2569]	-6.56	No significant similarity found	Unknown	2.09E-10
Q2_Reed_16wkturkeymuscle_08_O16	-6.47	NM_001006241	Gallus gallus ribosomal protein L3 (RPL3)	2.46E-12
Q1_Reed_18d_tembryo_04_O11_175	-6.38	NM_001040018	Gallus gallus olfactomedin-like 3 (OLFML3)	1.81E-11
Contig[2553]	-6.37	NM_205044	Gallus gallus matrix Gla protein (MGP)	1.69E-07
Contig[myosin_L2B_regulatory_li	-6.35	NM_001097526	Gallus gallus myosin, light chain 10, regulatory (MYL10)	9.88E-11
Q1_Reed_um_Mg_cDNA1_08_83C11	-6.25	NM_205114	Gallus gallus slow muscle troponin T (LOC396009)	1.51E-07
Contig[0486]	-6.20	NM_001030541	Gallus gallus periostin, osteoblast specific factor (POSTN)	3.72E-09
Q2_Reed_18d_tembryo_05_I06_089	-6.14	NM_204498	Gallus gallus Schwann cell-specific EGF-like repeat autocrine factor (LOC395159)	2.09E-10
Q2_Reed_16wkturkeymuscle_08_O16-C	-6.07	NM_001006241	Gallus gallus ribosomal protein L3 (RPL3)	1.23E-09

Contig[2562]	-5.90	XM_001231272	PREDICTED: Gallus gallus similar to PA2.26 antigen, transcript variant 1 (LOC768390)	3.91E-11
Contig[0098]	43.25	M74086	Gallus gallus fast myosin heavy chain mRNA, 3' UTR	2.12E-13
Contig[0054]	36.11	XM_414843	PREDICTED: Gallus gallus ribosomal protein L3-like (RPL3L)	6.36E-14
Contig[troponin_C_type_1_(slow)-C	35.03	NM_205133	Gallus gallus troponin C type 1 (slow) (TNNC1)	0
Q3_Reed_16wkturkeymuscle_08_H19	30.49	XM_418319	PREDICTED: Gallus gallus similar to Chain A, Crystal Structure Of S-Glutathiolated Carbonic Anhydrase Iii (LOC420208)	0
Q3_Reed_16wkturkeymuscle_05_D13	27.33	XM_001234683	PREDICTED: Gallus gallus similar to eEF1A2 binding protein (LOC771410)	0
Q3_Reed_16wkturkeymuscle_08_H21	25.71	AF210256	Meleagris gallopavo fast skeletal muscle troponin T mRNA	1.64E-11
Contig[0059]	25.49	NM_001013397	Gallus gallus myosin, heavy chain 6, cardiac muscle, alpha (MYH6)	0
Q1_Reed_1DPH_02_03_E13_197	16.50	XM_425039	PREDICTED: Gallus gallus fructose-1,6-bisphosphatase 2 (FBP2)	2.61E-08
Q3_Reed_16wkTurkeyMuscle_03_F21	16.42	XM_425101	PREDICTED: Gallus gallus hypothetical LOC427527 (LOC427527)	8.94E-09
Q1_Reed_16wkturkeyMuscle_04_I23	15.87	XM_422058	PREDICTED: Gallus gallus similar to Tubulin--tyrosine ligase-like protein 4 (LOC424209)	1.19E-13
Contig[2425]	15.79	NM_205417	Gallus gallus troponin I type 2 (skeletal, fast) (TNNI2)	8.36E-11
Contig[1199]	15.38	NM_205284	Gallus gallus lactate dehydrogenase A (LDHA)	5.25E-07
Contig[1412]	14.21	AF226597	Meleagris gallopavo fast skeletal muscle troponin T isoform 1e17	1.23E-09
Contig[2711]	14.01	XM_425039	PREDICTED: Gallus gallus fructose-1,6-bisphosphatase 2 (FBP2)	7.49E-10
Contig[troponin_C_type_1_(slow)	14.01	NM_205133	Gallus gallus troponin C type 1 (slow) (TNNC1)	0
Contig[0183]	13.91	NM_204922	Gallus gallus troponin T type 3 (skeletal, fast) (TNNT3)	2.77E-10
Q3_Reed_16wkTurkeyMuscle_04_B23	12.95	AF210256	Meleagris gallopavo fast skeletal muscle troponin T mRNA	8.89E-10
Q4_Reed_16wkturkeymuscle_07_H24	12.74	AF210256	Meleagris gallopavo fast skeletal muscle troponin T mRNA	2.62E-10

Contig[0137]	12.08	AF210256	Meleagris gallopavo fast skeletal muscle troponin T mRNA	2.98E-09
beta-RYR	11.81	EU177006	Meleagris gallopavo skeletal muscle ryanodine receptor isoform beta (beta-RYR)	4.31E-10
Contig[0247]	11.49	NM_204922	Gallus gallus troponin T type 3 (skeletal, fast) (TNNT3)	1.07E-09
Q4_Reed_1DPH_cDNA_03_F12_182	11.31	BX935600	Gallus gallus finished cDNA, clone ChEST83i13	2.88E-08
Contig[0156]	11.30	XM_416397	PREDICTED: Gallus gallus tubulin, alpha 8 (TUBA8)	2.48E-09
Contig[2281]	11.23	AF210256	Meleagris gallopavo fast skeletal muscle troponin T	1.76E-08
Q2_Reed_16wkturkeymuscle_05_O20	11.20	XM_415936	PREDICTED: Gallus gallus kinesin family member 21A (KIF21A)	0
Q4_Reed_16wkTurkeyMuscle_04_L10	11.11	AF226597	Meleagris gallopavo fast skeletal muscle troponin T isoform 1e17	6.71E-10
Q3_Reed_16wkturkeymuscle_05_F11	10.75	NM_001030768	Gallus gallus 2,3-bisphosphoglycerate mutase (BPGM)	1.05E-12
Q4_Reed_16wkTurkeyMuscle_01_N10	10.40	AF210256	Meleagris gallopavo fast skeletal muscle troponin T	1.79E-08
Q4_Reed_um_mg_cDNA1_04_87G11	10.35	NM_001030768	Gallus gallus 2,3-bisphosphoglycerate mutase (BPGM)	2.08E-11
Contig[1863]-C	10.31	AF210256	Meleagris gallopavo fast skeletal muscle troponin T	6.72E-09
Contig[1941]	10.21	AJ292038	Gallus gallus partial mRNA for MHC class II-associated invariant chain (CD74 gene)	2.28E-12
Contig[0213]	9.67	XM_424899	PREDICTED: Gallus gallus creatine kinase, mitochondrial 2 (sarcomeric) (CKMT2)	4.07E-09
