# Supplementary material for: Transcriptional profiling identifies differentially expressed genes in developing turkey skeletal muscle
Source: BMC Genomics. 2011 Mar 8;12:143. doi: 10.1186/1471-2164-12-143 (PMC3060885; doi:10.1186/1471-2164-12-143)
Supplement: Additional File 3 — Supplementary Table 2 (Table S2). Top down-regulated and up-regulated genes (RBC2:F) in Experiment 2 for the developmental stages 18de, 1d, and 16wk with fold changes, GenBank accession numbers, and putative annotations. [file 1471-2164-12-143-S3.PDF]

Supplementary Table 2. Top down-regulated and up-regulated genes (RBC2:F) in Experiment 2 for the developmental stages 18de, 1d, and 16wk with fold changes, GenBank accession numbers, and putative annotations.

**Array ID**	**Fold change: 18de**	**GenBank Accession #**	**Putative Annotation**	**FDR**
Q3_Reed_16wkturkeymuscle_08_H01	-4.61	NM_001010842	Gallus gallus heat shock protein 25 (HSP25)	0.105
HSP25	-2.77	NM_001010842	Gallus gallus heat shock protein 25 (HSP25)	0.096
HSP_70	-2.56	NM_001006685	Gallus gallus heat shock 70kDa protein 2 (HSPA2)	0.096
Q1_Reed_18d_tembryo_01_O01_015	-1.78	No significant similarity found	Unknown	0.018
Q2_Reed_18d_tembryo_02_K04_059	-1.74	NM_001012917	Gallus gallus WAS/WASL interacting protein family, member 1 (WIPF1)	0.021
Q1_Reed_um_mg_cDNA1_02_60D08	-1.72	CR524407	Gallus gallus finished cDNA, clone ChEST276c1	0.056
Q4_Reed_UM_Mg_cDNA1_07_90B12	-1.44	No significant similarity found	Unknown	0.105
Contig[1033]	-1.37	No significant similarity found	Unknown	0.096
Q1_Reed_UM_Mg_cDNA1_10_94F12	-1.32	DQ213088	Taeniopygia guttata clone 0058P0012D10 protein phosphatase 1 regulatory (inhibitor) subunit 14B-like	0.096
Q1_Reed_18d_tembryo_05_C19_291	-1.29	CR338727	Gallus gallus finished cDNA, clone ChEST109e12	0.096
Q4_Reed_18d_tembryo_07_N04_062	-1.27	XM_422146	PREDICTED: Gallus gallus similar to Low-density lipoprotein receptor-related protein 1B precursor (LRP-DIT) (LOC424301)	0.084
Q2_Reed_um_Mg_cDNA1_09_12D02	-1.27	No significant similarity found	Unknown	0.096
Q2_Reed_1DPH_cDNA_08_A06_081	-1.26	XM_415116	PREDICTED: Gallus gallus similar to SET domain-containing protein 8 (LOC416825)	0.096
Q1_Reed_um_mg_cDNA1_02_34B05	-1.26	XM_420892	PREDICTED: Gallus gallus SEC15-like 2 (S. cerevisiae) (SEC15L2)	0.084
Q3_Reed_18d_tembryo_06_J07_106	-1.25	AC145942	Gallus gallus BAC clone CH261-21I23 from chromosome unknown	0.084

Q3_Reed_1DPH_cDNA_05_F19_294	-1.25	No significant similarity found	Unknown	0.096
Q2_Reed_16wkturkeymuscle_09_G12	-1.25	No significant similarity found	Unknown	0.096
Q3_Reed_1DPH_cDNA_07_L17_268	-1.25	NM_204313	Gallus gallus histone deacetylase 4 (HDAC4)	0.105
Q3_Reed_1DPH_cDNA_10_L15_236	-1.24	CR352758	Gallus gallus finished cDNA, clone ChEST10c13	0.096
Q3_Reed_UM_Mg_cDNA1_07_06F01	-1.22	XM_001234302	PREDICTED: Gallus gallus similar to POL-like (LOC770983)	0.096
Q2_Reed_um_Mg_cDNA_01_48H06	-1.21	XM_416349	PREDICTED: Gallus gallus similar to KIAA1277 protein (LOC418115)	0.096
NFAT-distance-3	-1.20	XM_418906	nuclear factor of activated T cells-transcription factor C2	0.060
Q1_Reed_18d_tembryo_07_M17_269	-1.19	No significant similarity found	Unknown	0.096
Q3_Reed_18d_tembryo_04_B21_322	2.91	No significant similarity found	Unknown	0.018
Q2_Reed_um_mg_cDNA1_02_88H11	1.77	No significant similarity found	Unknown	0.105
Q1_Reed_um_mg_cDNA1_04_07G01	1.72	XM_415449	PREDICTED: Gallus gallus hypothetical LOC417166 (LOC417166)	0.105
Q3_Reed_16wkTurkeyMuscle_04_J23	1.70	XM_421623	PREDICTED: Gallus gallus annexin A7 (ANXA7)	0.096
Q3_Reed_18d_tembryo_05_L09_140	1.70	XM_415615	PREDICTED: Gallus gallus hypothetical LOC417352 (LOC417352)	0.084
Q3_Reed_1DPH_cDNA_01_P21_336	1.61	XM_420914	PREDICTED: Gallus gallus similar to Dhcr7 (LOC422982)	0.096
Q4_Reed_UM_Mg_cDNA1_10_60D08	1.59	XM_416866.2	PREDICTED: Gallus gallus similar to PP2A B subunit PR48 (LOC418670)	0.018
Q4_Reed_um_mg_cDNA1_04_69E09	1.59	NM_001004429	Gallus gallus transforming, acidic coiled-coil containing protein 3 (TACC3)	0.096
Q1_Reed_18d_tembryo_04_K03_043	1.56	XM_421626	PREDICTED: Gallus gallus helicase, lymphoid-specific (HELLS)	0.088
Q1_Reed_18d_tembryo_05_G19_295	1.54	XM_424490	PREDICTED: Gallus gallus Rac GTPase activating protein 1 (RACGAP1)	0.096

Contig[2719]	1.54	XM_423859	PREDICTED: Gallus gallus similar to Hypothetical protein BC001096 (LOC426196)	0.057
Q1_Reed_um_Mg_cDNA1_08_56H07	1.52	XM_424376	PREDICTED: Gallus gallus MCM4 minichromosome maintenance deficient 4 (S. cerevisiae) (MCM4)	0.096
Q4_Reed_um_Mg_cDNA_01_39G05	1.52	XM_417838	PREDICTED: Gallus gallus hypothetical LOC419690 (LOC419690)	0.016
Q4_Reed_18d_tembryo_05_H12_184	1.50	XM_414619	PREDICTED: Gallus gallus hypothetical LOC416300 (LOC416300)	0.096
Q1_Reed_18d_tembryo_05_C23_355	1.48	NM_205286.1	Gallus gallus lamin B1 (LMNB1)	0.019
Q1_Reed_18d_tembryo_07_I13_201	1.46	XM_418879	PREDICTED: Gallus gallus enhancer of zeste homolog 2 (Drosophila)(EZH2)	0.096
Q3_Reed_UM_Mg_cDNA1_06_34B05	1.45	XM_424421.2	PREDICTED: Gallus gallus similar to tripartite motif-containing 36 (LOC426811)	0.025
Contig[0469]	1.40	XM_417838	PREDICTED: Gallus gallus hypothetical LOC419690 (LOC419690)	0.091
Contig[2853]-C	1.40	XM_418179	PREDICTED: Gallus gallus hypothetical LOC420058 (LOC420058)	0.089
Q1_Reed_UM_Mg_cDNA1_05_85E11	1.40	No significant similarity found	Unknown	0.096
Q3_Reed_18d_tembryo_03_H09_136	1.40	NM_001006431	Gallus gallus plastin 3 (T isoform) (PLS3)	0.096
Q3_Reed_um_mg_cDNA1_02_36D05	1.39	XM_001234209	PREDICTED: Gallus gallus hypothetical protein LOC770891	0.060
Q1_Reed_18d_tembryo_02_I23_361	1.39	XM_415428.2	PREDICTED: Gallus gallus similar to sarcosine dehydrogenase (LOC417146)	0.006
Q4_Reed_um_Mg_cDNA1_09_41A06	1.39	L11659	Chicken alpha-1 type XIV collagen	0.060
Q1_Reed_1DPH_cDNA_02_A21_321	1.38	L11659	Chicken alpha-1 type XIV collagen	0.084
Q4_Reed_1DPH_cDNA_04_P04_064	1.37	NM_001030880	Gallus gallus chromosome 1 open reading frame 144 (C1orf144)	0.084
Q4_Reed_UM_Mg_cDNA1_05_10B02	1.37	NM_001079753	Gallus gallus protein regulator of cytokinesis 1 (PRC1)	0.096
Q1_Reed_UM_Mg_cDNA1_05_77E10	1.34	NM_001006276	Gallus gallus replication factor C (activator 1) 3, 38kDa (RFC3)	0.096

Q2_Reed_UM_Mg_cDNA1_10_86F11	1.34	L22152	Gallus gallus reverse transcriptase gene, 3'end	0.096
Q4_Reed_18d_tembryo_06_D02_020	1.31	NM_001031236	Gallus gallus soc-2 suppressor of clear homolog (C. elegans)(SHOC2)	0.056

**Array ID**	**Fold change: 1d**	**GenBank Accession #**	**Putative Annotation**	**FDR**
Contig[0822]	-2.06	AY393845	Gallus gallus calreticulin	0.094
Q2_Reed_16wkturkeymuscle_06_K06	-1.94	XM_001234985	PREDICTED: Gallus gallus aminopeptidase puromycin sensitive (NPEPPS)	0.088
Contig[1369]	-1.87	NM_205107	Gallus gallus collagen, type VI, alpha 1 (COL6A1)	0.094
Q1_Reed_UM_Mg_cDNA1_10_32H04	-1.74	XM_415995	PREDICTED: Gallus gallus leiomodin 2 (cardiac) (LMOD2)	0.060
Contig[2456]	-1.56	NM_204200	Gallus gallus aryl hydrocarbon receptor nuclear translocator (ARNT)	0.045
Contig[2723]	-1.54	BX929539	Gallus gallus finished cDNA, clone ChEST985c8	0.060
Contig[2528]	-1.49	XM_416438	PREDICTED: Gallus gallus similar to sarcospan-2 (LOC418211)	0.094
Q4_Reed_18d_tembryo_03_L12_188	-1.46	NM_001006537	Gallus gallus importin 13 (IPO13)	0.094
Q1_Reed_1DPH_cDNA_12_M23_365	-1.45	XM_419831	PREDICTED: Gallus gallus mannosidase, endo-alpha (MANEA)	0.030
Q1_Reed_18d_tembryo_07_A19_289	-1.43	NM_001006507	Gallus gallus protein phosphatase 2, regulatory subunit B, delta isoform (PPP2R2D)	0.075
Q3_Reed_16wkturkeymuscle_07_L09	-1.37	BX934673	Gallus gallus finished cDNA, clone ChEST387b16	0.094
Q2_Reed_1DPH_cDNA_12_O02_031	-1.35	No significant similarity found	Unknown	0.012
Q2_Reed_16wkturkeymuscle_08_O12	-1.34	NM_001012837	Gallus gallus NECAP endocytosis associated 2 (NECAP2)	0.045
Q4_Reed_um_Mg_cDNA1_09_57A08	-1.19	NM_001006148	Gallus gallus bromodomain containing 8 (BRD8)	0.056

IFN-alpha	1.21	EU937528	Gallus gallus breed white leghorn interferon alpha precursor (IFN-alpha)	0.030
Q1_Reed_18d_tembryo_07_E17_261	1.36	XM_416367	PREDICTED: Gallus gallus gonad expressed transcript (GET)	0.012
Q1_Reed_16wkturkeymuscle_06_K09	1.46	BX934685	Gallus gallus finished cDNA, clone ChEST385m1	0.094
Q3_Reed_1DPH_cDNA_10_P23_368	1.50	XM_419987	PREDICTED: Gallus gallus similar to MGC82892 protein (LOC421977)	0.045
Q3_Reed_UM_Mg_cDNA1_05_06F01	1.51	No significant similarity found	Unknown	0.037

Array ID	Fold change 16wk	GenBank Accession #	Putative Annotation	FDR
Q4_Reed_16wkTurkeyMuscle_02_P14	-3.21	NM_204535	Gallus gallus secreted phosphoprotein 1 (osteopontin, bone sialoprotein I, early T-lymphocyte activation 1) (SPP1)	0.0004
Q3_Reed_16wkTurkeyMuscle_04_P01	-2.73	No significant similarity found	Unknown	0.018
Q2_Reed_um_Mg_cDNA1_08_90B12	-1.72	XM_416459	PREDICTED: Gallus gallus similar to beta-parvin (LOC418235)	0.041
Q1_Reed_1DPH_cDNA_06_A09_129	-1.69	XM_422662	PREDICTED: Gallus gallus similar to MGC82396 protein (LOC424851)	0.041
Q1_Reed_18d_tembryo_04_E15_229	1.35	XM_419284	PREDICTED: Gallus gallus coiled-coil domain containing 104 (CCDC104)	0.104
Q1_Reed_16wkturkeymuscle_05_K21	1.37	NM_001006236	Gallus gallus solute carrier family 25 (mitochondrial carrier; phosphate carrier), member 3 (SLC25A3), nuclear gene encoding mitochondrial protein	0.065
Q3_Reed_UM_Mg_cDNA1_05_06F01	1.51	No significant similarity found	Unknown	0.041
Q1_Reed_1DPH_02_01_16H02	2.08	XM_001236937	PREDICTED: Gallus gallus similar to similar to 60 kDa heat shock protein, mitochondrial precursor (Hsp60) (60 kDa chaperonin) (CPN60) (Heat shock protein 60) (HSP-60) (Mitochondrial matrix protein P1) (HSP-65) (LOC777492)	0.007
